# A long-awaited taxogenomic investigation of the family *Halomonadaceae*

**DOI:** 10.3389/fmicb.2023.1293707

**Published:** 2023-11-16

**Authors:** Rafael R. de la Haba, David R. Arahal, Cristina Sánchez-Porro, Maria Chuvochina, Stijn Wittouck, Philip Hugenholtz, Antonio Ventosa

**Affiliations:** ^1^Department of Microbiology and Parasitology, Faculty of Pharmacy, University of Sevilla, Sevilla, Spain; ^2^Departament of Microbiology and Ecology, University of Valencia, Valencia, Spain; ^3^The University of Queensland, School of Chemistry and Molecular Biosciences, Australian Centre for Ecogenomics, St Lucia, QLD, Australia; ^4^Research Group Environmental Ecology and Applied Microbiology, Department of Bioscience Engineering, University of Antwerp, Antwerp, Belgium

**Keywords:** phylogenomics, signature genes, halophiles, taxonomic reclassification, genus delineation

## Abstract

The family *Halomonadaceae* is the largest family composed of halophilic bacteria, with more than 160 species with validly published names as of July 2023. Several classifications to circumscribe this family are available in major resources, such as those provided by the List of Prokaryotic names with Standing in Nomenclature (LPSN), NCBI Taxonomy, Genome Taxonomy Database (GTDB), and Bergey’s Manual of Systematics of Archaea and Bacteria (BMSAB), with some degree of disagreement between them. Moreover, regardless of the classification adopted, the genus *Halomonas* is not phylogenetically consistent, likely because it has been used as a catch-all for newly described species within the family *Halomonadaceae* that could not be clearly accommodated in other *Halomonadaceae* genera. In the past decade, some taxonomic rearrangements have been conducted on the *Halomonadaceae* based on ribosomal and alternative single-copy housekeeping gene sequence analysis. High-throughput technologies have enabled access to the genome sequences of many type strains belonging to the family *Halomonadaceae*; however, genome-based studies specifically addressing its taxonomic status have not been performed to date. In this study, we accomplished the genome sequencing of 17 missing type strains of *Halomonadaceae* species that, together with other publicly available genome sequences, allowed us to re-evaluate the genetic relationship, phylogeny, and taxonomy of the species and genera within this family. The approach followed included the estimate of the Overall Genome Relatedness Indexes (OGRIs) such as the average amino acid identity (AAI), phylogenomic reconstructions using amino acid substitution matrices customized for the family *Halomonadaceae*, and the analysis of clade-specific signature genes. Based on our results, we conclude that the genus *Halovibrio* is obviously out of place within the family *Halomonadaceae,* and, on the other hand, we propose a division of the genus *Halomonas* into seven separate genera and the transfer of seven species from *Halomonas* to the genus *Modicisalibacter*, together with the emendation of the latter. Additionally, data from this study demonstrate the existence of various synonym species names in this family.

## 1. Introduction

First described in 1988 to accommodate the genera *Halomonas* and *Deleya* based on 16S rRNA oligonucleotide cataloging, the family *Halomonadaceae* (Franzmann et al., 1988) is the largest bacterial family harboring halophilic microorganisms. Since then, 13 new genera with validly published names and one non-validly published name have been added to the family, as stated in the List of Prokaryotic names with Standing in Nomenclature (LPSN) (Parte et al., 2020). As of July 2023, it comprises 168 species with validly published names (according to the LPSN) that share the following features: Gram-stain-negative, non–endospore-forming rods or coccoid cells, chemo-organotrophic, aerobic or facultatively anaerobic, catalase-positive and oxidase-variable, and halophilic or halotolerant bacteria (Ventosa et al., 2021b). In accordance with the LPSN, this family belongs to the order *Oceanospirillales* (Garrity et al., 2005b), class *Gammaproteobacteria* (Garrity et al., 2005a), and other taxonomic sources indicate that it is most closely related to three genera belonging to the family *Oceanospirillaceae* (Garrity et al., 2005c): *Oceanospirillum*, *Marinospirillum*, or *Pseudospirillum*. The most widely used prokaryotic classification resources have different taxonomic opinions on this family. According to Bergey’s Manual of Systematics of Archaea and Bacteria (BMSAB), the most recent description of the family *Halomonadaceae* comprises 12 genera with validly published names: *Halomonas* (type genus), *Aidingimonas*, *Carnimonas*, *Chromohalobacter*, *Cobetia*, *Halotalea*, *Kushneria*, *Larsenimonas*, *Modicisalibacter*, *Pistricoccus*, *Salinicola*, and *Zymobacter* (Ventosa et al., 2021b), as well as one genus with an effectively published name, “*Phytohalomonas*” (Liu et al., 2020). As of July 2023, LPSN and NCBI Taxonomy also assign these 13 genera to the *Halomonadaceae* and 2 additional genera: *Halovibrio* and *Terasakiispira* in LPSN (Parte et al., 2020) and *Halovibrio* and *Salicola* in NCBI Taxonomy (Schoch et al., 2020), while Genome Taxonomy Database (GTDB) (Parks et al., 2018) release 08-RS214 comprises the 13 BMSAB genera together with the genus *Terasakiispira*, the 3 *Oceanospirillaceae* genera listed above, and an unnamed genus (TA22) based on environmental sequence data. GTDB also proposes the division of the genus *Halomonas* into five distinct genera (denoted with alphabetical suffixes) for a total of 22 genera in this family. Hence, currently, there is some disagreement on the circumscription of this family, which warrants further investigation.

For the present study, we followed the taxonomy proposed by BMSAB, but irrespective of the classification adopted, most genera of the family *Halomonadaceae* are well-defined and conserved, with the notable exception of the type genus of the family, *Halomonas*. *Halomonas* is the 15th largest genus across the prokaryotes in terms of the number of species with validly published names (121 as of July 2023) according to the LPSN.^1^ The species of this genus seldom form a monophyletic group and are instead scattered amongst the other genera of the family *Halomonadaceae* (de la Haba et al., 2010, 2012, 2014; Ventosa et al., 2021a,b). The polyphyly of *Halomonas* probably stems from the use of this genus as a default parent taxon for newly described members of the family that did not fall in any of the well-defined genera. Members of this genus exhibit a high degree of heterogeneity, as can be evidenced by their broad DNA G + C range (51.4–74.3 mol%), the widest across the family (Ventosa et al., 2021a). These observations point to the necessity of dividing the genus *Halomonas* and reclassifying some of its species.

After its description, three studies have proposed amendments to the family *Halomonadaceae*, accompanied by the inclusion of new genera into this family (Dobson and Franzmann, 1996; Ben Ali Gam et al., 2007; Ntougias et al., 2007). Additionally, the type genus of the family, *Halomonas*, has been amended on one occasion to accommodate nine new species in the genus (Dobson and Franzmann, 1996). Initially, a number of characteristic signatures for the 16S rRNA gene sequence were defined for members of this family (Dobson et al., 1993). Nevertheless, as new members have been described, it has been necessary to readapt the 16S rRNA gene sequence signatures (Ben Ali Gam et al., 2007; de la Haba et al., 2014). As whole-genome sequencing has become easier and cheaper, the analysis of clade-specific signature genes has been proposed as a tool to supplement the use of 16S rRNA gene sequence signatures (Zheng et al., 2020).

Several taxonomic studies have been carried out on the *Halomonadaceae*, some of them based on the 16S rRNA gene sequence analysis (Dobson et al., 1993; Mellado et al., 1995; Dobson and Franzmann, 1996), and the most recent focused on alternative phylogenetic markers, such as the 23S rRNA gene (Arahal et al., 2002; de la Haba et al., 2010) and multilocus sequence analysis based on single-copy housekeeping genes (de la Haba et al., 2012). Those approaches allowed, at that time, to establish the phylogenetic relationships among the members of this family and to propose taxonomic rearrangements accordingly. More than 10 years later, the number of species within the *Halomonadaceae* has doubled, and many of the type strains have a publicly available genome sequence. To the best of our knowledge, no studies specifically addressing the taxonomic status of the family *Halomonadaceae* in the genomic era have been conducted.

Therefore, in this study, we re-evaluate the genetic relationship, phylogeny, and taxonomy of the species and genera within the present family *Halomonadaceae*, as delineated in BMSAB, with the primary focus on its type genus. For this, we considered various overall genome relatedness indexes (OGRIs) based on average nucleotide identity (ANI), digital DNA–DNA hybridization (dDDH), average amino acid identity (AAI), core-protein average amino acid identity (cAAI), and the core-genome and GTDB phylogenies together with a signature gene analysis. To resolve the polyphyly within the genus *Halomonas*, we propose a division of the genus into seven different genera and the reclassification of several *Halomonas* species into the genus *Modicisalibacter*. Furthermore, the present study demonstrated the need to exclude the genus *Halovibrio* from the family *Halomonadaceae* and the existence of nine synonym species across this family.

## 2. Materials and methods

### 2.1. Genome retrieval and sequencing of type strains

The databases scanned for genomic data were NCBI Assembly,^2^ NCBI Sequence Read Archive (SRA),^3^ JGI Genome Portal,^4^ ATCC Genome Portal,^5^ and Global Catalogue of Type Strain (Shi et al., 2021). A total of 147 genome sequences of distinct type strains of species of the family *Halomonadaceae* (as per the BMSAB resource) and the genera *Halovibrio* and *Terasakiispira* were available in those public databases at the beginning of this study. Since some databases searched contained redundant data, the final set of genomes used in this research was only retrieved from the NCBI Assembly and NCBI SRA databases. One out of those 147 available genomes was discarded due to a failed quality check according to GTDB (Parks et al., 2018), while another marked as contaminated and excluded from RefSeq was finally retained after the verification of its contamination and completeness in GTDB, giving a total number of 146 genome sequences (Supplementary Table 1). All except one of the downloaded genomic data were assembled either at contig, scaffold, or chromosome level. The remaining one was only available in the NCBI SRA database, and thus, only raw reads could be retrieved, which were processed as indicated below.

To make our study as comprehensive as possible, we sequenced the genomes of an additional 17 type strains of species of *Halomonadaceae* that were not publicly available when the analyses were designed and performed (Supplementary Table 1). Seven out of those type strains were sequenced and assembled by the Global Catalogue of Microorganisms (Shi et al., 2021) as part of the type strain sequencing project (Wu and Ma, 2019). The genomic DNA of the other 10 type strains, retrieved from culture collections, was extracted and purified as described elsewhere (Galisteo et al., 2022) and further sequenced by Novogene Europe (Cambridge, United Kingdom) on the Illumina NovaSeq PE150 platform after library preparation of genomic material using the Novogene NGS DNA Library Prep Set (Cat. No. PT004). Raw reads were quality trimmed and filtered with BBTools v.38.44 (Bushnell, 2020) and further assembled with SPAdes v3.15.2 (option “—isolate”) (Prjibelski et al., 2020).

For all the retrieved or obtained sequences, assembly quality, and basic statistics were estimated with QUAST v.2.3 (Gurevich et al., 2013). Genome sequence completeness and contamination were verified by using CheckM v.1.1.5 (Parks et al., 2015). Prodigal v.2.6.3 (Hyatt et al., 2010) was employed to extract the translated coding sequences from the assembled genomes, and Prokka v.1.14.6 (Seemann, 2014) was employed for the automated annotation. When more than one genome sequence was available for the same type strain, we selected the one with a better assessment of contig number, completeness, contamination, 16S rRNA gene sequence presence and length, and the presence of 20 essential amino acids coded by distinct tRNA-coding genes, resulting in a final set of 161 genomes of type strains of the family *Halomonadaceae*.

### 2.2. Pangenome and core-genome inference

The complete pangenome of the selected set of genome sequences was calculated with Anvi’o suite v.7.1 (Eren et al., 2021), using BLASTP+ (Camacho et al., 2009) as the amino acid sequence similarity search program. Specifically, the “anvi-pan-genome” script was used with default values except that the MCL inflation parameter was set to 5 (in order to increase the sensitivity of the Markov Cluster Algorithm) (van Dongen, 2008), and the minimum percent identity between two amino acid sequences was set to 40%. The pangenome database containing orthologous gene clusters was employed to extract the gene family presence/absence matrix (“anvi-compute-functional-enrichment-in-pan” script) as well as the translated single-copy core genes present in at least 90% of the analyzed genomes, denoted as “core90” (“anvi-get-sequences-for-gene-clusters” script).

The “core90” proteins were individually aligned with MUSCLE v.3.8.1551 (default parameters) (Edgar, 2004). Gappy columns (>50% gaps) from each alignment were removed and further pruned using the chi2 statistic to filter out the 20% least conserved columns, as implemented in the “alignment_pruner.pl” Perl script.^6^ Final alignments were concatenated whenever necessary using AMAS (Borowiec, 2016).

### 2.3. Estimation of amino acid substitution models

Model-based phylogenetic analyses of protein sequences strongly rely on amino acid substitution models, which are generally summarized in a 20-by-20 replacement matrix, designated as *Q* matrices. Since those matrices are computationally very expensive to estimate due to their high parametrization, they are not usually estimated during a phylogenetic analysis but selected from a pre-estimated set of *Q* matrices using model selection software (Minh et al., 2021).

With the aim of constructing very reliable phylogenies, in this study, we estimated empirical general time-reversible *Q* matrices customized for the family *Halomonadaceae* derived from multiple sequence alignments of selected gene sets. For that purpose, QMarker software (Minh et al., 2021), which follows a maximum-likelihood approach, was used. Two gene datasets were chosen to generate *Halomonadaceae*-specific *Q* matrices: the “core90” set and the “bac120” set (120 single-copy bacterial proteins used by GTDB taxonomy) (Parks et al., 2018).

### 2.4. Phylogenomic reconstructions and overall genome relatedness indexes

The trimmed and concatenated “core90” protein alignment was used to infer the maximum-likelihood phylogeny of the family *Halomonadaceae* with the IQ-TREE v.2.2.0 program (Minh et al., 2020), using the custom *Q*_core90 reversible matrix as the substitution model combined with profile mixture models C10–C60 (Quang et al., 2008). The best-fit model parameters (mixture models, amino acid frequencies, and rate heterogeneity across sites) were determined with ModelFinder (Kalyaanamoorthy et al., 2017) according to Bayesian Information Criterion (BIC) (Schwarz, 1978). Branch support was estimated via 1,000 ultrafast bootstrap approximations (Hoang et al., 2018). Normalization of taxonomic ranks at the genus level according to relative evolutionary divergence (RED) was carried out with PhyloRank.^7^

A second phylogenomic tree including type and non-type strains as well as metagenome-assembled genomes (MAGs) was constructed to assess the cluster stability when new genome sequences are added to the tree and to analyze the relationships between members of the family *Halomonadaceae* and other closely related genera of the order *Oceanospirillales* (i.e., *Halospina*, *Marinospirillum*, *Oceanospirillum*, and *Pseudospirillum*). This second tree was based on the “bac120” protein set retrieved from GTDB Release 202 data files using GTDB-Tk v. 1.7.0 (Chaumeil et al., 2019). However, unlike the original GTDB-Tk pipeline that randomly selected 42 columns per marker to reduce computational requirements, we obtained the full-length multiple sequence alignments for each protein, which were later trimmed and concatenated as explained above for the “core90” dataset. Phylogenetic inference was carried out as stated for the first tree, but the *Q*_bac120 reversible matrix was used instead. Arrangements and visualization of both trees were accomplished by using ARB v.7.0 (Westram et al., 2011).

Commonly used OGRIs for taxonomic purposes were pairwise calculated among the analyzed genome set. ANI metrics were estimated with PyANI v.0.2.10 (Pritchard et al., 2016) using the ANIb method (i.e., BLASTN+ to align 1,020-bp fragments of the input sequences) (Goris et al., 2007). The genome-to-genome distance calculator (GGDC) webserver was used to infer dDDH relatedness according to formula 2 (Meier-Kolthoff et al., 2013). AAI values were determined using the “aai.rb” script from the Enveomics collection (Rodriguez-R and Konstantinidis, 2016). Since horizontal gene transfer (HGT) events can distort the molecular clock of bacterial evolution (Novichkov et al., 2004) and can, thus, impact the pairwise AAI values (Zheng et al., 2020), we calculated an additional genome relatedness index denoted as cAAI, similar to AAI but based on the protein sequences of core orthologous gene families, which are less impacted by HGT. For that purpose, the previously defined “core90” protein dataset (unaligned and untrimmed) was selected to calculate cAAI values with the “aai.rb” script.

### 2.5. Representation of clade-specific signature genes

The gene family presence/absence matrix inferred from the pangenome was used to detect “signature genes,” which are defined as gene families exclusive to specific phylogenetic clades (phylogroups), that is, gene families present in all species of a clade and absent in all other species (Zheng et al., 2020). Several phylogroups were investigated, aimed at testing different taxonomic arrangements and splitting into the family *Halomonadaceae*. For each phylogroup, a representative species was chosen, and the remaining species under study were assigned to the phylogroup of the representative species with whom they shared a most recent common ancestor.

The signature gene UpSet plots were generated by gathering gene families with the same presence/absence pattern across the analyzed genomes, ruling out two types of trivial patterns: gene families present in a single genome (singleton genes) and gene families detected in all genomes (core genes). The patterns were then displayed in descending order of frequency (number of gene families). R packages “tidyverse” v.1.2.1 (Wickham et al., 2019) and “tidygenomes” v.0.1.3,^8^ as well as other R functions and scripts,^9^ were used for data processing and visualization of gene family presence/absence and signature genes.

## 3. Results and discussion

### 3.1. Core-genome phylogenomics suggest several rearrangements in the family *Halomonadaceae*

**Figure 1:**
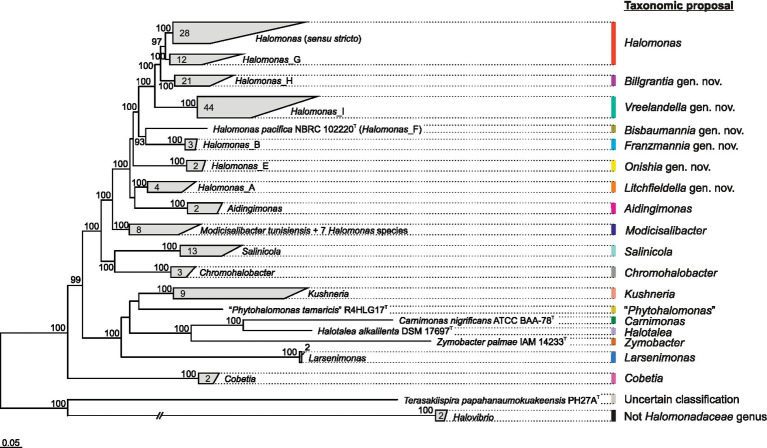
Clade collapsed maximum-likelihood phylogenomic tree based on the concatenation of the translated sequence of 189 single-copy genes shared by at least 90% of the members of the family *Halomonadaceae* under study (“core90” set). The genus *Pistricoccus* is missing because no representative genome sequences from type strains of this genus were recovered. The number of species comprised within each clade is displayed. Ultrafast bootstrap values ≥70% are shown above the branches. Bar, 0.05 changes per position.

A genome-based analysis was carried out aimed at shedding light on the relationships among species and genera of the family *Halomonadaceae*. The pangenome of the 161 genome sequences, which included type strains of the species belonging to the family *Halomonadaceae*, consisted of 608,808 genes organized into 59,992 orthologous gene clusters. Only 55 single-copy core gene clusters were detected across all genomes, so a more relaxed set of 189 orthologous genes shared by at least 90% of the genomes (“core90”) encompassing a total of 23,924 residues after alignment and trimming was selected to calculate a *Halomonadaceae*-specific *Q* reversible matrix (*Q*_core90; Supplementary Table 2) and to infer a phylogenomic tree (Figure 1 and Supplementary Figure 1). The best-fit model selected by ModelFinder according to the BIC criterion to construct the tree was *Q*_core90 + C60 + F + R10. The resulting tree showed that, apart from the genus *Halomonas*, all the remaining genera within this family formed monophyletic groups. Noteworthy, the genera *Terasakiispira* and primarily *Halovibrio* were by far the most distantly related to the other genera within *Halomonadaceae*, suggesting that they might not belong to this family (see below).

Given the polyphyletic origin of the genus *Halomonas* and the heterogeneous characteristics of the species it harbors, we evaluated several proposals to split this genus into several genera with the following constraints: (i) the new proposed genera must be monophyletic; (ii) they must be supported by a 100% bootstrap value when possible; and (iii) they must fall within the relative evolutionary divergence (RED) interval defined for the rank of genus (Parks et al., 2018). The first attempt (*proposal I*) was to divide the genus *Halomonas* into eight phylogroups, five of them denoted according to GTDB taxonomy as *Halomonas* (*sensu stricto*), *Halomonas*_A, *Halomonas*_B, *Halomonas*_E, and *Halomonas*_F, and three newly proposed phylogroups designated as *Halomonas*_G, *Halomonas*_H, and *Halomonas*_I, as well as to transfer the species *Halomonas coralii*, *Halomonas ilicicola*, *Halomonas muralis*, *Halomonas radicis*, *Halomonas xianhensis*, and *Halomonas zincidurans* to the genus *Modicisalibacter*. Subsequent proposals were similar to this but included merging some of the previous phylogroups: *proposal II* = *Halomonas* (*sensu stricto*) + *Halomonas*_G; *proposal III* = *Halomonas* (*sensu stricto*) + *Halomonas*_G + *Halomonas*_H; *proposal IV* = *Halomonas* (*sensu stricto*) + *Halomonas*_G + *Halomonas*_H + *Halomonas*_I. It should be noted that *proposal II* involved the formation of a phylogroup (*Halomonas* + *Halomonas*_G) supported “only” by a 97% bootstrap, which does not meet the desired constraints indicated above but is high enough to consider this option of grouping. To select the best-fitting scenario for this genus reclassification among the above proposals, we performed further comparative genomic analyses.

### 3.2. Assessment on the basis of AAI and cAAI values does not provide sound clues to split the genus *Halomonas*

**Figure 2:**
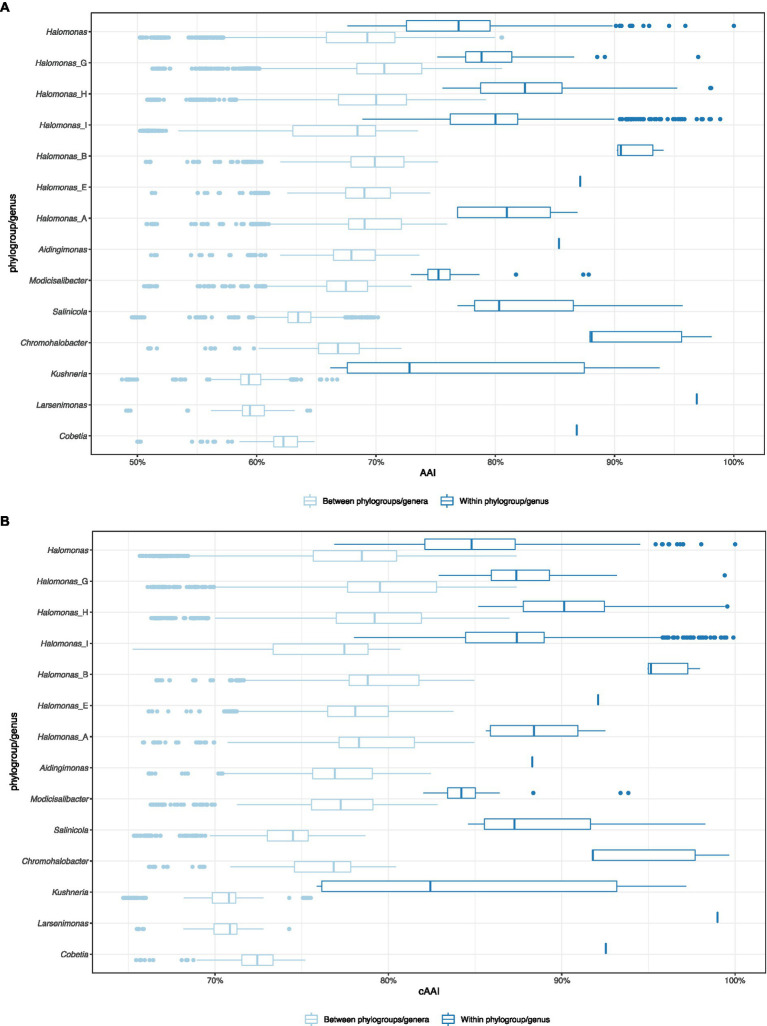
Box plot of the pairwise intra-clade and inter-clade AAI **(A)** and cAAI **(B)** values for the potential phylogroups/genera in the family *Halomonadaceae*, according to proposal I.

To evaluate the proposed phylogroups under the four scenarios, we plotted the all-*vs*-all AAI and cAAI results clustered by the phylogroup while distinguishing between intra-genus and inter-genus values (Figure 2). Theoretically, a well-delimited genus or phylogroup should not display any overlap between intra-clade and inter-clade AAI/cAAI values. As shown in Figure 2, all the currently existing genera within the family *Halomonadaceae* harboring more than a single species except *Halomonas* (*sensu stricto*) (i.e., *Aidingimonas*, *Salinicola*, *Chromohalobacter*, *Kushneria*, *Larsenimonas*, and *Cobetia*), as well as the new phylogroups shared by the four proposals (*Halomonas*_B, *Halomonas*_F, *Halomonas*_E, *Halomonas*_A, and the enlarged *Modicisalibacter*) showed minimal or no overlap between the inter- and intra-cluster AAI and cAAI values, confirming their clear separation and exclusivity as different genera within this family.

By contrast, the intra-group AAI and cAAI values largely overlapped with their inter-group counterparts for the phylogroups *Halomonas*, *Halomonas*_G, *Halomonas*_H, and *Halomonas*_I (*proposal I*), indicating their heterogeneity as well as the lack of clear differentiation among them. No significant overlap reduction was observed after the consecutive merger of these phylogroups (*proposals II*, *III*, and *IV*), although *proposals II* and *III* led to slightly decreased overlap (Supplementary Figures 2–4). Based on the above observations, we believe that AAI and cAAI data may be useful for the delineation of most of the genera of the family *Halomonadaceae*, but they are not reliable for fine phylogroup demarcation within the genus *Halomonas*. The lowest intra-clade AAI and cAAI values for the current genera and *proposals I*, *II*, and *III* were 66.2 and 75.9%, respectively, while the cutoffs for *proposal IV* were 65.5 and 73.2%, respectively. These threshold values might be useful for future genus circumscription within the family *Halomonadaceae*.

### 3.3. Signature genes support the inclusion of *Halomonas*_G into the genus *Halomonas*

Since AAI and cAAI values did not allow us to determine the best of the four proposals to split the genus *Halomonas*, we analyzed whether the currently existing genera and suggested phylogroups can be defined by clade-specific signature genes. For that purpose, we examined the gene family presence/absence matrix extracted from the pangenome of the family *Halomonadaceae* to detect gene families present in all genomes of a certain group but lacking in all other genomes. Signature genes may be the result of a shared evolutionary history within a phylogenetic clade, or they could be acquired by horizontal gene transfer reflecting common lifestyles, ecologies, and physiological properties (Zheng et al., 2020). Therefore, signature genes enable estimates of the evolutionary forces that shaped the cluster, and, thus, they might be of help to opt for one of the proposals over the others.

**Figure 3:**
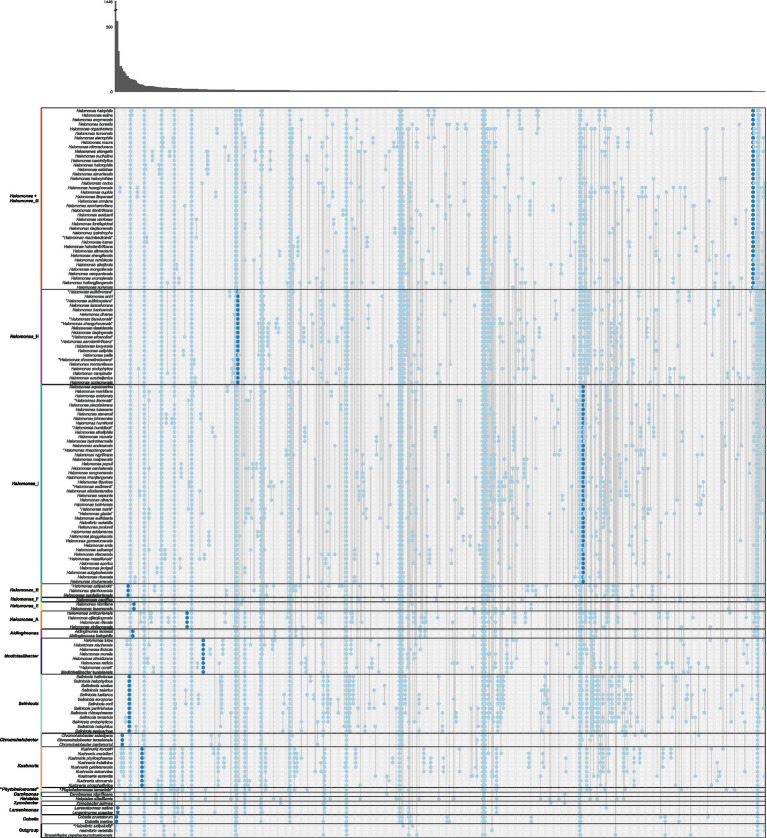
Gene family presence/absence patterns inferred from the pangenome of the family *Halomonadaceae* arranged according to *proposal II*. Each column represents a gene family pattern, where presence is indicated with a dot in the corresponding species. The absolute number of gene families that conform to each pattern is visualized in the marginal bar plot at the top. Separations between phylogroups/genera are indicated with horizontal black lines and the representative species of each phylogroup/genus is highlighted in bold. Genes that were present in all genomes of a clade and in none of the genomes outside of that clade, denoted as “signature genes,” are displayed in dark blue; other genes are shown in light blue. Patterns of presence in a single species or all species are not shown. The species *Halovibrio salipaludis*, *Halovibrio variabilis*, and *Terasakiispira papahanaumokuakeensis* were not considered members of the *Halomonadaceae* and were only used as an “outgroup”.

Except for *Halomonas*, all the current genera of the family *Halomonadaceae* according to the BMSAB classification containing more than a single species, together with *Halomonas*_B, *Halomonas*_E, *Halomonas*_A, the newly proposed phylogroups *Halomonas*_H and *Halomonas*_I, and the enlarged *Modicisalibacter*, were well supported by signature genes ranging from 5 to 548 (Figure 3 and Supplementary Figure 5). As can be expected, phylogroups/genera with a smaller number of species showed, generally, a larger number of signature genes; nevertheless, the impact of the clade size is limited, as can be evidenced from larger genera, such as *Salinicola* and *Kushneria*, displaying a relatively large number of signature genes. Considering that singleton genes (those present in a single genome) were omitted from the analysis, it was impossible to evaluate the signature genes harbored by phylogroups/genera containing only one species (i.e., *Halomonas*_F, “*Phytohalomonas*,” *Carnimonas*, *Halotalea*, and *Zymobacter*). Interestingly, no signature genes were identified for phylogroups *Halomonas* (*sensu stricto*) and *Halomonas*_G, which *a priori* dismisses *proposal I*. However, when merging these phylogroups (*proposal II*), a signature gene was detected (Figure 3). Although it is a single signature gene, it is conserved across a large number of genomes (40), comprising the phylogroups *Halomonas* and *Halomonas*_G and, therefore, it likely reflects a common evolutionary history of both phylogroups providing an important hint into the delineation of the genus *Halomonas*. *Proposal III*, which considers the merging of phylogroups *Halomonas*, *Halomonas*_G, and *Halomonas*_H, was also endorsed by a single signature gene (Supplementary Figure 5); however, *proposal II* is favored over *proposal III* due to the fact that phylogroup *Halomonas*_H is very well supported by 21 signature genes and, thus, it is preferable to keep it as a separate cluster. The last proposal, *proposal IV*, consisting of the fusion of phylogroups *Halomonas*, *Halomonas*_G, *Halomonas*_H, and *Halomonas*_I, can also be rejected because the combined clade did not show any signature genes (Supplementary Figure 5) and, in addition, phylogroup *Halomonas*_I was supported by five signature genes.

### 3.4. GTDB-based phylogeny is consistent with *proposal II* and delineates the family *Halomonadaceae*

Although signature gene analyses endorsed *proposal II* as the most reliable one to split the genus *Halomonas*, this proposal involves the creation of a cluster with “only” a 97% branch bootstrap, as stated above. While this support value cannot be regarded as too low, we attempted to establish a long-term and trustworthy classification of the family for which only 100% supported phylogroups are preferred. This concern raises questions about the suitability of *proposal II*, making it necessary to provide some additional evidence to accept or reject it.

**Figure 4:**
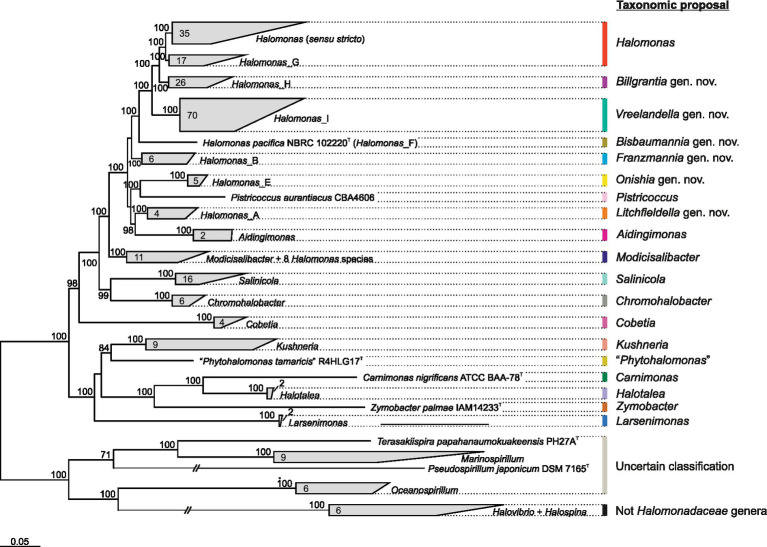
GTDB-derived maximum-likelihood phylogenomic tree based on the concatenation of 120 single-copy bacterial proteins (“bac120” set) showing the relationships among members of the family *Halomonadaceae* and related taxa. The type strain of the species *Pistricoccus aurantiacus* is missing because no genome sequences from this strain could be recovered. The number of species in each cluster is displayed inside wedges. Ultrafast bootstrap values ≥70% are shown above the branches. Bar, 0.05 changes per amino acid position.

Accordingly, an alternative family-specific *Q* time-reversible matrix (*Q*_bac120; Supplementary Table 3) and phylogenomic tree (Figure 4) were calculated on the basis of the “bac120” marker protein dataset used to infer the bacterial GTDB taxonomy (Parks et al., 2018), comprising a total of 32,354 aligned columns after trimming. These ubiquitous single-copy proteins have been identified as being suitable for phylogenetic inference (Parks et al., 2017) and may yield better resolved trees than those obtained by using the almost entire set of core proteins (“core90” set). In fact, some of the “core90” non-curated protein-coding genes might have been laterally transferred or undergone homologous recombination (Stott and Bobay, 2020) or might lack congruent phylogenetic signals or sufficient homology to make comparisons valid and conclusive (Wu et al., 2013; Capella-Gutierrez et al., 2014). In addition, genomes from non-type strains and MAGs were also included in this complementary reconstruction to evaluate tree topology preservation.

The topology of the “bac120”-based tree (Figure 4) recovered the same phylogroups observed in the “core90”-based phylogeny (Figure 1). Although it is well-known that the selection of different phylogenetic markers can result in different topologies (Capella-Gutierrez et al., 2014; Ankenbrand and Keller, 2016; Tsai et al., 2019), in our analysis, the bias associated with marker choice did not affect the delineation of genera and phylogroups, demonstrating the stability of the obtained genome-based trees with independence of the protein set used for their inference. A good agreement between core-genome and “bac120” phylogenies has been previously demonstrated for the taxa of the *Pseudomonadaceae* (Lalucat et al., 2020), which is another family in the class *Gammaproteobacteria*. The inclusion of 82 additional GTDB representative genomes from non-type strains and MAGs of members of the family *Halomonadaceae*, as well as genomes from the closest genera of the order *Oceanospirillales*, did not alter the retrieved clades either (Figure 4). The most remarkable difference between our “core90” and “bac120” trees is the stronger support in the latter of the branch collapsing phylogroups *Halomonas* (*sensu stricto*) and *Halomonas*_G (corresponding to *proposal II*). Hence, this 100% bootstrap value recovered in the “bac120” tree after merging both phylogroups, together with the signature gene analysis, enables *proposal II* as the most appropriate to prune the genus *Halomonas* to the species comprising phylogroups *Halomonas* (*sensu stricto*) and *Halomonas*_G, while transferring the remaining species to six new genera and to the already described genus *Modicisalibacter*.

Concerning the taxonomic status of the family *Halomonadaceae*, the “bac120”-based phylogeny evidenced that all 12 genera currently affiliated to this family according to BMSAB, together with the non-validly published genus name “*Phytohalomonas*,” formed a monophyletic group of microorganisms (Figure 4), whose shared feature, which was at the same time differential to the closest related genera, was their halophily or halotolerance. The genus *Halovibrio*, also considered a member of the family *Halomonadaceae* by LPSN, was intimately related to the genus *Halospina*, belonging to the family *Hahellaceae*; ^10^^,^^11^ whereas the genus *Terasakiispira*, included into the *Halomonadaceae* by both LPSN and GTDB resources, was rather a taxon within the family *Oceanospirillaceae* given its closest relationship to the genera *Marinospirillum*, *Oceanospirillum*, and *Pseudospirillum* (Figure 4). GTDB taxonomy (release 08-RS214) has suggested the incorporation of these three genera into the family *Halomonadaceae*, even if they harbor marine (slight) halophiles in contrast to the majority of moderately halophilic bacteria characteristic of the family *Halomonadaceae*. Therefore, it becomes clear that the genus *Halovibrio* should be kept apart from the family *Halomonadaceae*, while further investigation including genomic, phylogenomic, and phenotypic comparisons is required to delineate the taxonomic affiliation of the genera *Terasakiispira*, *Marinospirillum*, *Oceanospirillum*, and *Pseudospirillum* at the family level.

### 3.5. Heterotypic synonyms revealed by ANI and dDDH values

**Figure 5:**
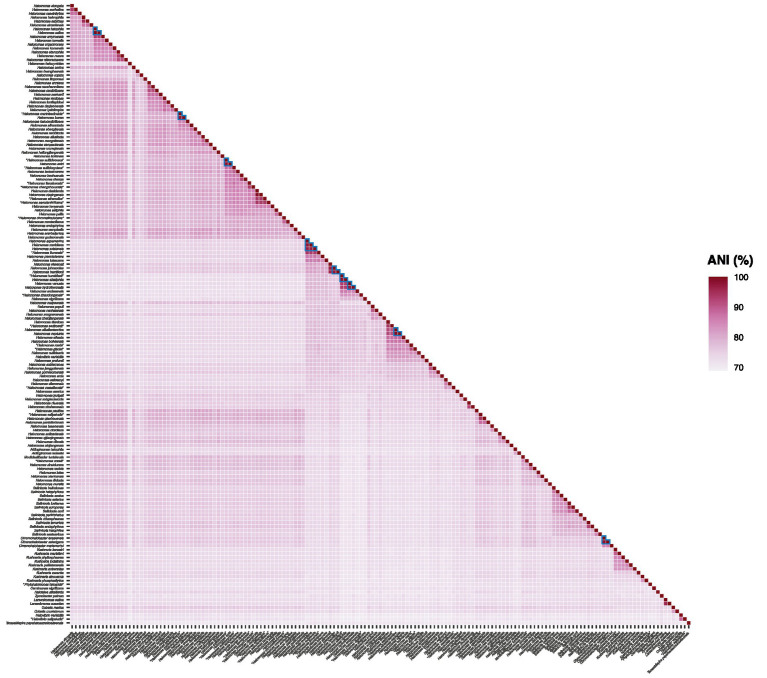
ANI values (%) from pairwise whole-genome comparisons between the type strains of members of the family *Halomonadaceae*. The ANI percentages are expressed as a heatmap in purple color. Values supporting the proposed synonym species names are surrounded by a blue triangle.

Phylogenomic inference using both the “core90” and the “bac120” protein sets revealed the presence of some very closely related species pairs and even one set of three among the studied taxa that might be considered cases of heterotypic synonymy. To verify those hypotheses, ANI (Figure 5) and dDDH relatedness indexes were estimated between the genomes in question. It is widely accepted that cutoff values for species delineation based on these genomic indexes are 95–96% for ANI (Goris et al., 2007; Richter and Rossello-Mora, 2009; Chun and Rainey, 2014) and 70% for dDDH (Auch et al., 2010). Therefore, two or more strains can be considered to belong to the same species if both ANI values are ≥96% and dDDH values are ≥70%. According to this criterion, our results confirmed the existence of the following sets of heterotypic synonyms (ANI and dDDH values in parenthesis, respectively): *Chromohalobacter israelensis* – *Chromohalobacter salexigens* (98.1 and 83.6%); *Halomonas alkaliphila* – “*Halomonas humidisoli*” (97.7 and 80.5%); *Halomonas antri* – “*Halomonas sulfidivorans*” (97.7 and 80.2%); *Halomonas aquamarina* – *Halomonas meridiana* – *Halomonas axialensis* (96.5–97.5% and 71.2–79.1%); *Halomonas halophila* – *Halomonas salina* (100 and 100%); *Halomonas icarae* – “*Halomonas marinisediminis*” (97.4 and 79.2%); *Halomonas neptunia* – *Halomonas alkaliantarctica* (98.9 and 91.1%); *Halomonas hamiltonii* – *Halomonas johnsoniae* (97.3 and 76.8%); and *Halomonas venusta* – *Halomonas hydrothermalis* (96.8 and 73.0%).

## 4. Taxonomic conclusions

The comparative genomic analyses (phylogenomics, OGRI, and signature genes) conducted among the type strains of the species of the family *Halomonadaceae* allow us to propose several taxonomic rearrangements within this family:

- The genus *Halomonas* comprises the following species: *H. elongata* (type species), *H. aestuarii*, *H. alimentaria*, *H. alkalicola*, *H. almeriensis*, *H. borealis*, *H. campaniensis*, *H. caseinilytica*, *H. cerina*, *H. cupida*, *H. daqiaonensis*, *H. denitrificans*, *H. eurihalina*, *H. fontilapidosi*, *H. halmophila*, *H. halocynthiae*, *H. halodenitrificans*, *H. halophila* (synonym *H. salina*), *H. heilongjiangensis*, *H. huangheensis*, *H. icarae* (synonym “*H. marinisediminis*”), *H. koreensis*, *H. korlensis*, *H. litopenaei*, *H. lysinitropha*, *H. maura*, *H. mongoliensis*, *H. nitroreducens*, *H. organivorans*, *H. ramblicola*, *H. sabkhae*, *H. saccharevitans*, *H. shengliensis*, *H. smyrnensis*, *H. stenophila*, *H. urmiana*, *H. urumqiensis*, and *H. ventosae*.
- The following species are transferred to the genus *Modicisalibacter*: “*Halomonas coralii*,” *Halomonas ilicicola*, *Halomonas lutea*, *Halomonas muralis*, *Halomonas radicis*, *Halomonas xianhensis*, and *Halomonas zincidurans*, as “*Modicisalibacter coralii*,” *Modicisalibacter ilicicola*, *Modicisalibacter luteus*, *Modicisalibacter muralis*, *Modicisalibacter radicis*, *Modicisalibacter xianhensis*, and *Modicisalibacter zincidurans*, respectively. Therefore, it has been necessary to extend the description of the genus *Modicisalibacter*, as indicated below.
- The remaining analyzed species of the genus *Halomonas* are reclassified into new genera belonging to the family *Halomonadaceae* as follows, where the type species of each genus is highlighted in bold, according to the rule of priority of publication:

- Genus *Vreelandella* (corresponding to the phylogroup *Halomonas*_I), including the species *H. alkaliphila* (synonym “*H. humidisoli*”), *H. andesensis*, ***H. aquamarina*** (synonyms *H. meridiana* and *H. axialensis*), *H. arcis*, *H. azerica*, *H. boliviensis*, “*H. glaciei*,” *H. gomseomensis*, *H. hamiltonii* (synonym *H. johnsoniae*), *H. janggokensis*, *H. jeotgali*, “*H. lionensis*,” *H. lutescens*, *H. malpeensis*, “*H. maris*,” “*H. massiliensis*,” *H. nanhaiensis*, *H. neptunia* (synonym *H. alkaliantarctica*), *H. nigrificans*, *H. olivaria*, *H. piezotolerans*, *H. populi*, *H. profundi*, *H. rituensis*, *H. salicampi*, “*H. sedimenti*,” *H. songnenensis*, *H. stevensii*, *H. subglaciescola*, *H. subterranea*, *H. sulfidaeris*, *H. titanicae*, *H. utahensis*, *H. venusta* (synonym *H. hydrothermalis*), *H. vilamensis*, *H. zhanjiangensis*, “*H. zhaodongensis*,” and *H. zhuhanensis*.
- Genus *Bisbaumannia* (corresponding to the phylogroup *Halomonas*_F), including the single species ***H. pacifica***.
- Genus *Billgrantia* (corresponding to the phylogroup *Halomonas*_H), including the species “*H. aerodenitrificans*,” *H. antri* (synonym “*H. sulfidivorans*”), *H. azerbaijanica*, *H. bachuensis*, *H. campisalis*, “*H. chromatireducens*,” ***H. desiderata*** (synonym *H. daqingensis*), *H. diversa*, *H. endophytica*, “*H. ethanolica*,” *H. gudaonensis*, *H. kenyensis*, *H. lactosivorans*, “*H. montanilacus*,” *H. pellis*, *H. saliphila*, “*H. sulfidoxydans*,” “*H. tianxiuensis*,” and “*H. zhangzhouensis*.”
- Genus *Franzmannia* (corresponding to the phylogroup *Halomonas*_B), including the species ***H. pantelleriensis***, *H. qiaohouensis*, and “*H. salipaludis*.”
- Genus *Litchfieldella* (corresponding to the phylogroup *Halomonas*_A), including the species ***H. anticariensis***, *H. qijiaojingensis*, *H. rifensis*, and *H. xinjiangensis*.
- Genus *Onishia* (corresponding to the phylogroup *Halomonas*_E), including the species *H. niordiana* and ***H. taeanensis***.

- The genus *Halovibrio* should be excluded from the family *Halomonadaceae*. According to GTBD, it should be transferred to the family *Oleiphilaceae*.
- The following species sets are considered heterotypic synonyms (the species name holding priority in the application of the International Code of Nomenclature of Prokaryotes (Oren et al., 2023) is listed in first place): *Chromohalobacter israelensis* – *Chromohalobacter salexigens*; *Halomonas alkaliphila* – “*Halomonas humidisoli*”; *Halomonas antri* – “*Halomonas sufidivorans*”; *Halomonas aquamarina* – *Halomonas meridiana* – *Halomonas axialensis*; *Halomonas halophila* – *Halomonas salina*; *Halomonas hamiltonii* – *Halomonas johnsoniae*; *Halomonas icarae* – “*Halomonas marinisediminis*”; *Halomonas neptunia* – *Halomonas alkaliantarctica*; and *Halomonas venusta* - *Halomonas hydrothermalis*.
- The species whose genome sequence was not publicly available or could not be sequenced when this study was accomplished, together with the new species described after the finalization of this research, should be further studied to determine their correct placement according to the taxonomy proposed here or if they may constitute new genera within the family *Halomonadaceae*.

Moreover, we strongly recommend the use of the clade-specific amino acid substitution matrices *Q*_core90 and *Q*_bac120, empirically calculated for the family *Halomonadaceae*, for future phylogenomic studies within this family instead of the pre-computed *Q* matrices generally selected by model selection software.

### Description of *Vreelandella* gen. nov.

*Vreelandella* (Vree.land.el’la N.L. fem. dim. n. *Vreelandella*, named after Russell Vreeland, American scientist who described the genus *Halomonas* and studied halophilic microorganisms for over 40 years).

Cells are Gram-staining-negative rods, 0.3–1.9 × 0.5–6.0 μm in size, aerobic or facultatively anaerobic, and mostly motile. Endospores are not formed. Catalase-positive and oxidase-variable. Colonies are translucent, beige, black, cream, glistening-colored, ochre, orange, pale-pink, reddish-brown, white, or yellow pigmented. Slightly to moderately halophilic, growing at 0–27% (w/v) NaCl, with optimal growth at 0–15% (w/v) NaCl. Mesophilic or psychrotolerant, thriving at −5–60°C, showing optimal growth at 20–37°C. Alkaliphilic or alkalitolerant, growing at pH values in the range of 5.0–12.0, with optimal growth at pH 7.0–10.0. Chemo-organotrophic. Nitrate reduction is variable. The major respiratory quinones are Q9, Q8, and Q6. The major fatty acids are C_16:0_, C_16:1_*ω*6*c*/C_16:1_*ω*7*c*/iso-C_15:0_ 2-OH, C_18:1_*ω*6*c*/C_18:1_*ω*7*c*, C_19:0_ cyclo *ω*8*c*, C_12:0_ 3-OH, C_17:0_ cyclo, C_10:0_, iso-C_16:1_*ω*7*c* 2-OH, and C_18:0_. The major polar lipids are diphosphatidylglycerol, phosphatidylglycerol, and phosphatidylethanolamine.

The DNA G + C content ranges between 52.1 and 63.8 mol%.

The genus *Vreelandella* belongs to the family *Halomonadaceae*. The type species is *Vreelandella aquamarina*.

### Description of *Vreelandella aquamarina* comb. nov.

*Vreelandella aquamarina* (a.qua.ma.ri’na. L. fem. n. *aqua*, water; L. masc. adj. *marinus*, of the sea, marine; N.L. fem. adj. *aquamarina*, pertaining to seawater).

Basonym: “*Achromobacter aquamarinus*” ZoBeAll and Upham 1944.

Homotypic synonyms: *Halomonas aquamarina* (ZoBell and Upham 1944) Dobson and Franzmann, 1996; *Deleya aquamarina* (ZoBell and Upham 1944) Akagawa and Yamasato 1989; *Alcaligenes aquamarinus* (ZoBell and Upham 1944) Hendrie *et al.* 1974 (Approved Lists 1980).

Cells are Gram-stain-negative rods, 0.6–1.9 × 1.0–4.5 μm in size, with rounded ends, occurring singly or in doublets, facultatively anaerobic, and motile by means of peritrichous or lateral flagella. Endospores are not formed. Catalase and oxidase are positive. Colonies are convex, smooth, circular, entire, whitish or cream-colored, and 2 mm in diameter after 2 days of incubation on Marine Agar 2,216 at 20°C. Slightly to moderately halophilic, growing at 0–30% (w/v) NaCl, with optimal growth at 0.5–10% (w/v) NaCl. Psychrotolerant, thriving at −1–47°C, showing optimal growth at 20–40°C. Alkalitolerant, growing at pH values in the range of 5.0–12.0, with optimal growth at pH 7.0–8.0. Chemo-organotrophic. Negative for Simmons’ citrate utilization, indole production, methyl red and Voges–Proskauer tests, lysine decarboxylase, and β-galactosidase. Aesculin, casein, gelatin, and DNA are not hydrolyzed. Variable for nitrate reduction to nitrite, H_2_S production, urease, hydrolysis of starch and Tween 80, ornithine decarboxylase, phosphatase, and phenylalanine deaminase. Produces acid but no gas from glucose. Does not ferment glycerol or xylose. Utilizes L-arabinose, D-fructose, D-glucose, maltose, sucrose, and fumarate as sole carbon and energy sources. The major respiratory quinone is Q9. The major fatty acids are C_16:0_, C_18:1_*ω*7*c*, C_16:1_*ω*7*c*/iso-C_15:0_ 2-OH, C_19:0_ cyclo *ω*8*c*, and C_12:0_ 3-OH. The major polar lipids are diphosphatidylglycerol, phosphatidylglycerol, and phosphatidylethanolamine.

The DNA G + C content is 56.7–57.0 mol%.

The type strain is 558^T^ = ATCC 14400^T^ = BCRC 12878^T^ = CCUG 16157^T^ = CECT 5000^T^ = CGMCC 1.2324^T^ = CIP 105454^T^ = DSM 30161^T^ = IAM 12550^T^ = KCTC 22193^T^ = LMD 73.17^T^ = LMG 2853^T^ = NCIMB 557^T^. The genome size of the type strain is 3.50 Mbp, and its DNA G + C content is 56.7 mol%. Isolated from seawater (Pacific Ocean).

Type strain genome sequence accession number: GCA_900110265.1.

Type strain 16S rRNA gene sequence accession number: AJ306888.

*Halomonas meridiana* and *Halomonas axialensis* should be regarded as heterotypic synonyms of *Vreelandella aquamarina*.

### Description of *Vreelandella venusta* comb. nov.

*Vreelandella venusta* (ve.nus’ta. L. fem. adj. *venusta*, lovely, beautiful).

Basonym: *Alcaligenes venustus* Baumann *et al.* 1972 (Approved Lists 1980).

Homotypic synonyms: *Halomonas venusta* (Baumann *et al.* 1972) Dobson and Franzmann 1996; *Deleya venusta* (Baumann *et al.* 1972) Baumann *et al.* 1983.

Cells are Gram-stain-negative rods, 1.5 × 2.0–3.0 μm in size, occurring singly or in doublets, aerobic or facultatively anaerobic, and motile by means of peritrichous flagella. Accumulate β-polyhydroxybutyrate as an intracellular reserve product. Endospores are not formed. Catalase and oxidase are positive. Colonies are round, smooth, and yellow or cream-colored. Slightly to moderately halophilic, growing at 0–22% (w/v) NaCl, with optimal growth at 0.5–7% (w/v) NaCl. Psychrotolerant, thriving at 2–40°C, showing optimal growth at 30°C. Alkalitolerant, growing at pH values in the range of 5.0–12.0, with optimal growth at pH 7.0–8.0. Chemo-organotrophic. Positive for nitrate reduction to nitrite. Negative for indole production and methyl red and Voges–Proskauer tests. DNA is hydrolyzed, but alginate, casein, chitin, gelatin, starch, and Tween 80 are not. Variable for H_2_S production, lysine decarboxylase, ornithine decarboxylase, and phenylalanine deaminase. Acid production from D-glucose is variable, but negative from L-arabinose, D-fructose, D-galactose, lactose, maltose, D-mannose, sucrose, D-trehalose, D-xylose, or glycerol. Utilizes D-glucose, glycerol, acetate, butyrate, citrate, fumarate, DL-malate, propionate, L-glutamate, L-lysine, and L-proline as sole carbon (nitrogen) and energy sources. The major respiratory quinone is Q9. The major fatty acids are C_16:0_, C_18:1_*ω*7*c*, C_16:1_*ω*7*c*/iso-C_15:0_ 2-OH, C_19:0_ cyclo *ω*8*c*, and C_12:0_ 3-OH. The major polar lipids are diphosphatidylglycerol, phosphatidylglycerol, and phosphatidylethanolamine.

The DNA G + C content is 52.6–53.2 mol%.

The type strain is 86^T^ = ATCC 27125^T^ = CCUG 16063^T^ = CIP 103201^T^ = DSM 4743^T^ = JCM 20634^T^ = LMG 3445^T^ = NBRC 102221^T^. The genome size of the type strain is 4.27 Mbp, and its DNA G + C content is 52.6 mol%. Isolated from seawater (off the coast of Oahu, Hawaii, United States).

Type strain genome sequence accession number: GCA_007989605.1.

Type strain 16S rRNA gene sequence accession number: AJ306894.

*Halomonas hydrothermalis* should be regarded as a heterotypic synonym of *Vreelandella venusta*.

### Description of *Vreelandella subglaciescola* comb. nov.

*Vreelandella subglaciescola* (sub.gla.ci.es’co.la. L. prep. *sub-*, under, below; L. fem. n. *glacies*, ice; L. masc. n. suff. *-cola*, dweller; N.L. fem. n. *subglaciescola*, dwelling below the ice).

Basonym: *Halomonas subglaciescola* Franzmann *et al.* 1987.

The description is as given in the proposal of the basonym (Franzmann et al., 1987), with the following addition. The genome size of the type strain is 3.11 Mbp. The DNA G + C content is 60.8 mol%.

Isolated from saline water from Organic Lake, Antarctica.

The type strain is ACAM 12^T^ = ATCC 43668^T^ = CIP 104042^T^ = DSM 4683^T^ = IAM 14167^T^ = JCM 21045^T^ = LMG 8824^T^ = NBRC 14766^T^ = UQM 2926^T^.

Type strain genome sequence accession number: GCA_900142895.1.

Type strain 16S rRNA gene sequence accession number: AJ306892.

### Description of *Vreelandella neptunia* comb. nov.

*Vreelandella neptunia* (nep.tu’ni.a. L. fem. adj. *neptunia*, Neptunian, pertaining to Neptunus, the Roman god of the sea).

Basonym: *Halomonas neptunia* Kaye *et al.* 2004.

Cells are Gram-stain-negative rods, 0.5–1.0 × 1.0–3.0 μm in size, occurring singly or in doublets, aerobic or facultatively anaerobic, and motile by means of peritrichous flagella. Produces exopolysaccharides and accumulates glycine-betaine but not β-polyhydroxybutyrate. Endospores are not formed. Oxidase-positive, but catalase-variable. Colonies are round, smooth, and cream-colored. Slightly to moderately halophilic, growing at 0.5–27% (w/v) NaCl, with optimal growth at 2–10% (w/v) NaCl. Psychrotolerant, thriving at −1–37°C, showing optimal growth at 30°C. Alkalitolerant, growing at pH values in the range of 5.0–12.0, with optimal growth at pH 7.0–9.0. Chemo-organotrophic. Positive for nitrate reduction to nitrite, but nitrite is not reduced. Negative for Simmons’ citrate utilization, H_2_S production, methyl red and Voges–Proskauer tests, lysine decarboxylase, ornithine decarboxylase, and phenylalanine deaminase. Able to synthesize α-glucosidase. Casein, DNA, gelatin, starch, and Tween 80 are not hydrolyzed. Variable for indole production. Acid production from D-galactose and D-glucose is positive, but negative from L-arabinose, D-fructose, lactose, maltose, D-mannose, sucrose, D-trehalose, D-xylose, or glycerol. Utilizes D-cellobiose, D-fructose, D-galactose, D-glucose, maltose, D-ribose, sucrose, D-trehalose, glycerol, and acetate as sole carbon and energy sources. The only respiratory quinone is Q9. The major fatty acids are C_18:1_*ω*7*c*, C_16:0_, C_16:1_*ω*7*c*, C_19:0_ cyclo *ω*8*c*, and C_12:0_ 3-OH. The major polar lipids are phosphatidylglycerol, diphosphatidylglycerol, and phosphatidylethanolamine.

The DNA G + C content is 54.8–55.0 mol%.

The type strain is Eplume1^T^ = ATCC BAA-805^T^ = CCM 7107^T^ = CECT 5815^T^ = DSM 15720^T^. The genome size of the type strain is 4.93 Mbp, and its DNA G + C content is 55.0 mol%. Isolated from a deep-sea hydrothermal plume (NE Pacific Ocean, Juan de Fuca Ridge).

Type strain genome sequence accession numbers: GCA_030409295.1 and GCA_019903445.1.

Type strain 16S rRNA gene sequence accession number: AF212202.

*Halomonas alkaliantarctica* should be regarded as a heterotypic synonym of *Vreelandella neptunia*.

### Description of *Vreelandella sulfidaeris* comb. nov.

*Vreelandella sulfidaeris* (sul.fid.ae’ris. N.L. neut. n. *sulfidum*, sulfide; L. neut. n. *aes*, ore; N.L. gen. n. *sulfidaeris*, from sulfide ore).

Basonym: *Halomonas sulfidaeris* Kaye *et al.* 2004.

The description is as given in the proposal of the basonym (Kaye et al., 2004), with the following addition. The genome size of the type strain is 4.48 Mbp. The DNA G + C content is 53.7 mol%.

Isolated from a deep-sea sulfide rock (NE Pacific Ocean, Juan de Fuca Ridge).

The type strain is Esulfide1^T^ = ATCC BAA-803^T^ = CCM 7108^T^ = CECT 5817^T^ = DSM 15722^T^.

Type strain genome sequence accession number: GCA_007182875.1.

Type strain 16S rRNA gene sequence accession number: AF212204.

### Description of *Vreelandella boliviensis* comb. nov.

*Vreelandella boliviensis* (bo.li.vi.en’sis. N.L. fem. adj. *boliviensis*, from Bolivia, relating to the country where the bacteria were isolated).

Basonym: *Halomonas boliviensis* Quillaguamán *et al.* 2004.

The description is as given in the proposal of the basonym (Quillaguamán et al., 2004), with the following addition. The genome size of the type strain is 4.21 Mbp. The DNA G + C content is 54.7 mol%.

Isolated from the soil around a Bolivian hypersaline lake.

The type strain is LC1^T^ = ATCC BAA-759^T^ = DSM 15516^T^.

Type strain genome sequence accession numbers: GCA_000236035.1 and GCA_002265845.1.

Type strain 16S rRNA gene sequence accession number: AY245449.

### Description of *Vreelandella utahensis* comb. nov.

*Vreelandella utahensis* (u.ta.hen’sis. N.L. fem. adj. *utahensis*, referring to Utah).

Basonym: *Halomonas utahensis* Sorokin and Tindall, 2006.

The description is as given in the proposal of the basonym (Sorokin and Tindall, 2006), with the following addition. The genome size of the type strain is 3.73 Mbp. The DNA G + C content is 55.8 mol%.

Isolated from surface water from the North Arm of Great Salt Lake (United States).

The type strain is isolate III^T^ = ATCC 49240^T^ = CECT 5286^T^ = CIP 105504^T^ = DSM 3051^T^ = IAM 14440^T^ = JCM 21223^T^ = NBRC 102410^T^.

Type strain genome sequence accession number: GCA_007991175.1.

Type strain 16S rRNA gene sequence accession number: AJ306893.

### Description of *Vreelandella gomseomensis* comb. nov.

*Vreelandella gomseomensis* (gom.se.om.en’sis. N.L. fem. adj. *gomseomensis*, referring to Gomseom in Anmyeondo, from where the first strains were isolated).

Basonym: *Halomonas gomseomensis* Kim *et al.* 2007.

The description is as given in the proposal of the basonym (Kim et al., 2007), with the following addition. The genome size of the type strain is 3.73 Mbp. The DNA G + C content is 59.8 mol%.

Isolated from saline water of the Gomseom solar saltern in Anmyeondo (Korea).

The type strain is M12^T^ = CIP 109897^T^ = DSM 18042^T^ = KCTC 12662^T^.

Type strain genome sequence accession number: GCA_031451645.1.

Type strain 16S rRNA gene sequence accession number: AM229314.

### Description of *Vreelandella janggokensis* comb. nov.

*Vreelandella janggokensis* (jang.gok.en’sis. N.L. fem. adj. *janggokensis*, referring to Janggok in Anmyeondo, from where the first strains were isolated).

Basonym: *Halomonas janggokensis* Kim *et al.* 2007.

The description is as given in the proposal of the basonym (Kim et al., 2007), with the following addition. The genome size of the type strain is 3.87 Mbp. The DNA G + C content is 57.3 mol%.

Isolated from saline water of the Janggok solar saltern in Anmyeondo (Korea).

The type strain is M24^T^ = CIP 109896^T^ = DSM 18043^T^ = KCTC 12663^T^.

Type strain genome sequence accession number: GCA_031451615.1.

Type strain 16S rRNA gene sequence accession number: AM229315.

### Description of *Vreelandella arcis* comb. nov.

*Vreelandella arcis* (ar’cis. L. gen. n. *arcis*, of a height, summit, or peak, referring to the isolation of the organism from a salt lake on the Qinghai-Tibet Plateau).

Basonym: *Halomonas arcis* Xu *et al.* 2007.

The description is as given in the proposal of the basonym (Xu et al., 2007), with the following addition. The genome size of the type strain is 4.14 Mbp. The DNA G + C content is 55.9 mol%.

Isolated from the water of a salt lake located in Altun Mountain on the Qinghai-Tibet Plateau (China).

The type strain is AJ282^T^ = CGMCC 1.6494^T^ = DSM 23549^T^ = JCM 14607^T^ = LMG 23978^T^.

Type strain genome sequence accession number: GCA_900103865.1.

Type strain 16S rRNA gene sequence accession number: EF144147.

### Description of *Vreelandella subterranea* comb. nov.

*Vreelandella subterranea* (sub.ter.ra’ne.a. L. fem. adj. *subterranea*, underground, subterranean, referring to the isolation of the organism from the subterranean brines).

Basonym: *Halomonas subterranea* Xu *et al.* 2007.

The description is as given in the proposal of the basonym (Xu et al., 2007), with the following addition. The genome size of the type strain is 3.73 Mbp. The DNA G + C content is 58.0 mol%.

Isolated from water of a subterranean saline well at Si-Chuan Basin (China).

The type strain is ZG16^T^ = CIP 109673^T^ = CGMCC 1.6495^T^ = DSM 23550^T^ = JCM 14608^T^ = LMG 23977^T^.

Type strain genome sequence accession number: GCA_900111305.1.

Type strain 16S rRNA gene sequence accession number: EF144148.

### Description of *Vreelandella alkaliphila* comb. nov.

*Vreelandella alkaliphila* (al.ka.li’phi.la. N.L. neut. n. *alkali*, the ashes of saltwort [al-qaliy]; N.L. masc. adj. *philus* [from Gr. masc. adj. *philos*], friend, loving; N.L. fem. adj. *alkaliphila*, loving alkaline media).

Basonym: *Halomonas alkaliphila* Romano *et al.* 2007.

Cells are Gram-stain-negative rods, 0.3–0.7 × 0.5–2.6 μm in size, aerobic, and motile or non-motile. Produces exopolysaccharides and accumulates β-polyhydroxybutyrate, ectoine, and glycine-betaine. Catalase and oxidase are positive. Colonies are circular, wet, convex, and beige or cream-colored. Moderately halophilic, growing at 0–25% (w/v) NaCl, with optimal growth at 4–10% (w/v) NaCl. Mesophilic, thriving at 5–50°C, showing optimal growth at 30–37°C. Alkaliphilic or alkalitolerant, growing at pH values in the range of 5.0–12.0, with optimal growth at pH 7.5–9.0. Chemo-organotrophic. Positive for nitrate reduction to nitrite and tyrosine decomposition. Negative for H_2_S production. Hippurate and pectin are hydrolyzed, but casein, gelatin, phenylalanine, starch, Tween 80, and tyrosine are not. Variable for urease production. Utilizes D-glucose, D-galactose, D-fructose, sucrose, D-maltose, D-cellobiose, D-trehalose, glycerol, and acetate as sole carbon and energy sources. The major respiratory quinones are Q9, Q8, and Q6. The major fatty acids are C_18:1_*ω*6*c*/C_18:1_*ω*7*c*, C_16:0_, C_16:1_*ω*6*c*/C_16:1_*ω*7*c*, and C_18:0_. The major polar lipids are phosphatidylethanolamine, diphosphatidylglycerol, and phosphatidylglycerol.

The DNA G + C content is 52.5–52.6 mol%.

The type strain is 18bAG^T^ = ATCC BAA-953^T^ = DSM 16354^T^. The genome size of the type strain is 4.10 Mbp, and its DNA G + C content is 52.6 mol%. Isolated from a salt pool in Campania (Italy).

Type strain genome sequence accession number: GCA_016107625.1.

Type strain 16S rRNA gene sequence accession number: AJ640133.

### Description of *Vreelandella zhanjiangensis* comb. nov.

*Vreelandella zhanjiangensis* (zhan.ji.ang.en’sis. N.L. fem. adj. *zhanjiangensis*, pertaining to Zhanjiang, a city in China near where the sample was collected).

Basonym: *Halomonas zhanjiangensis* Chen *et al.*, 2009.

The description is as given in the proposal of the basonym (Chen et al., 2009), with the following addition. The genome size of the type strain is 4.06 Mbp. The DNA G + C content is 54.5 mol%.

Isolated from a sea urchin, *Hemicentrotus pulcherrimus*, South China Sea, tidal flat of Naozhou Island near Zhanjiang (China).

The type strain is JSM 078169^T^ = CCTCC AB 208031^T^ = DSM 21076^T^ = KCTC 22279^T^.

Type strain genome sequence accession number: GCA_000377665.1.

Type strain 16S rRNA gene sequence accession number: FJ429198.

### Description of *Vreelandella stevensii* comb. nov.

*Vreelandella stevensii* (ste.ven’si.i. N.L. gen. n. *stevensii*, of Stevens, named after Dr. David A. Stevens, a physician/epidemiologist who isolated and characterized the first strains).

Basonym: *Halomonas stevensii* Kim *et al.* 2010.

The description is as given in the proposal of the basonym (Kim K. K. et al., 2010), with the following addition. The genome size of the type strain is 3.69 Mbp. The DNA G + C content is 60.3 mol%.

Isolated from the blood of a renal care patient at California, San Jose, Santa Clara Valley Medical Center (United States).

The type strain is S18214^T^ = DSM 21198^T^ = KCTC 22148^T^.

Type strain genome sequence accession number: GCA_000275725.1.

Type strain 16S rRNA gene sequence accession number: AM941388.

### Description of *Vreelandella hamiltonii* comb. nov.

*Vreelandella hamiltonii* (ha.mil.to’ni.i. N.L. gen. n. *hamiltonii*, of Hamilton, named after Dr. John R. Hamilton, a microbiologist who isolated and characterized the first strains).

Basonym: *Halomonas hamiltonii* Kim *et al.* 2010.

The description is as given in the proposal of the basonym (Kim K. K. et al., 2010), with the following addition. Cells are 0.7–1.0 × 1.5–4.0 μm in size and motile with lateral or lateral/polar flagella. Optimal growth occurs at sea-salt concentrations of 0–7.5%, w/v. Grown on cetrimide agar. Nitrate reduction to nitrite, the Voges–Proskauer test, and urease are variable. Utilization of adipate, D-galactose, malonate, L-lysine, DL-isoleucine, and L-valine as sole sources of carbon (nitrogen) and energy is variable. Acid production from L-arabinose, D-fucose, D-galactose, methyl α-D-glucoside, glycerol, D-mannitol, and melezitose is variable. Susceptibility to neomycin, penicillin G, and chloramphenicol is strain-dependent. The DNA G + C content is 60.1 mol%.

The type strain is W1025^T^ = DSM 21196^T^ = KCTC 22154^T^. The genome size of the type strain is 3.93 Mbp, and its DNA G + C content is 60.1 mol%. Isolated from the blood of a dialysis machine drain at California, San Jose, Santa Clara Valley Medical Center (USA).

Type strain genome sequence accession number: GCA_014651775.1.

Type strain 16S rRNA gene sequence accession number: AM941396.

*Halomonas johnsoniae* should be regarded as a heterotypic synonym of *Vreelandella hamiltonii*.

### Description of *Vreelandella andesensis* comb. nov.

*Vreelandella andesensis* (an.de.sen’sis. N.L. fem. adj. *andesensis*, pertaining to the Andes).

Basonym: *Halomonas andesensis* Guzmán *et al.* 2010.

The description is as given in the proposal of the basonym (Guzmán et al., 2010), with the following addition. The genome size of the type strain is 3.91 Mbp. The DNA G + C content is 52.1 mol%.

Isolated from water from saline lake Laguna Colorada (22° 12’ S 67° 49’ W), 4,300 m above sea level (Bolivia).

The type strain is LC6^T^ = CCUG 54844^T^ = DSM 19434^T^ = LMG 24243^T^.

Type strain genome sequence accession number: GCA_003989795.1.

Type strain 16S rRNA gene sequence accession number: EF622233.

### Description of *Vreelandella titanicae* comb. nov.

*Vreelandella titanicae* (ti.tan’ic.ae. N.L. fem. n. *titanica*, the ship Titanic; N.L. gen. fem. n. *titanicae*, of or from the ship Titanic).

Basonym: *Halomonas titanicae* Mann *et al.* 2010.

The description is as given in the proposal of the basonym (Sánchez-Porro et al., 2010), with the following addition. The genome size of the type strain is 5.34 Mbp. The DNA G + C content is 54.6 mol%.

Isolated from the rusticles of the RMS Titanic wreck.

The type strain is BH1^T^ = ATCC BAA-1257^T^ = CECT 7585^T^ = DSM 22872^T^ = JCM 16411^T^ = LMG 25388^T^.

Type strain genome sequence accession number: GCA_000336575.1.

Type strain 16S rRNA gene sequence accession number: FN433898.

### Description of *Vreelandella vilamensis* comb. nov.

*Vreelandella vilamensis* (vi.la.men’sis. N.L. fem. adj. *vilamensis*, pertaining to Laguna Vilama, Jujuy, Argentina).

Basonym: *Halomonas vilamensis* Menes *et al.* 2011.

The description is as given in the proposal of the basonym (Menes et al., 2011), with the following addition. The genome size of the type strain is 3.47 Mbp. The DNA G + C content is 55.2 mol%.

Isolated from the sediment of hypersaline lake Laguna Vilama (22° 35’ S 66° 55’ W, 4,600 m above sea level) at Andean Puna desert, Jujuy (Argentina).

The type strain is SV325^T^ = DSM 21020^T^ = LMG 24332^T^.

Type strain genome sequence accession number: GCA_031451755.1.

Type strain 16S rRNA gene sequence accession number: EU557315.

### Description of *Vreelandella jeotgali* comb. nov.

*Vreelandella jeotgali* (je.ot.ga’li. N.L. gen. n. *jeotgali*, of jeotgal, a traditional Korean fermented seafood).

Basonym: *Halomonas jeotgali* Kim *et al.* 2011.

The description is as given in the proposal of the basonym (Kim M.-S. et al., 2010), with the following addition. The genome size of the type strain is 2.85 Mbp. The DNA G + C content is 62.9 mol%.

Isolated from jeotgal, a traditional Korean fermented seafood.

The type strain is Hwa^T^ = JCM 15645^T^ = KCTC 22487^T^.

Type strain genome sequence accession number: GCA_000334215.1.

Type strain 16S rRNA gene sequence accession number: EU909458.

### Description of *Vreelandella nanhaiensis* comb. nov.

*Vreelandella nanhaiensis* (nan.hai.en’sis. N.L. fem. adj. *nanhaiensis*, pertaining to Nanhai, a sea in South China where the sample was collected).

Basonym: *Halomonas nanhaiensis* Long *et al.* 2013.

The description is as given in the proposal of the basonym (Long et al., 2013), with the following addition. The genome size of the type strain is 4.03 Mbp. The DNA G + C content is 54.4 mol%.

Isolated from a sample of marine sediment at a depth of 310 m (74°52′35” S 163°53′03″ E), South China Sea.

The type strain is YIM M 13059^T^ = CCTCC AB 2012911^T^ = DSM 25561^T^ = JCM 18142^T^.

Type strain genome sequence accession number: GCA_003990185.1.

Type strain 16S rRNA gene sequence accession number: JX870002.

### Description of *Vreelandella olivaria* comb. nov.

*Vreelandella olivaria* (o.li.va’ri.a. L. fem. adj. *olivaria*, of or belonging to olives, related to olive-processing effluents from where the type strain was isolated).

Basonym: *Halomonas olivaria* Amouric *et al.* 2014.

The description is as given in the proposal of the basonym (Amouric et al., 2014), with the following addition. The genome size of the type strain is 5.00 Mbp. The DNA G + C content is 55.3 mol%.

Isolated from salted olive-processing effluents from an evaporation pond (Morocco).

The type strain is TYRC17^T^ = CCUG 53850 B^T^ = DSM 19074^T^.

Type strain genome sequence accession number: GCA_004295565.1.

Type strain 16S rRNA gene sequence accession number: DQ645593.

### Description of *Vreelandella songnenensis* comb. nov.

*Vreelandella songnenensis* (song.nen.en’sis. N.L. fem. adj. *songnenensis*, pertaining to Songnen Plain, north-east China, where the type strain was isolated).

Basonym: *Halomonas songnenensis* Jiang *et al.* 2014.

The description is as given in the proposal of the basonym (Jiang et al., 2014), with the following addition. The genome size of the type strain is 3.69 Mbp. The DNA G + C content is 59.1 mol%.

Isolated from saline and alkaline soil in an oilfield (46° 36′ 05.36” N 124° 55′ 00.36″ E), Songnen Plain (China).

The type strain is NEAU-ST10-39^T^ = CGMCC 1.12152^T^ = DSM 25870^T^.

Type strain genome sequence accession number: GCA_003002925.1.

Type strain 16S rRNA gene sequence accession number: JQ762289.

### Description of *Vreelandella salicampi* comb. nov.

*Vreelandella salicampi* (sa.li.cam’pi. L. masc. n. *sal*, salt; L. masc. n. *campus*, field; N.L. gen. n. *salicampi*, of a salt field).

Basonym: *Halomonas salicampi* Lee *et al.* 2015.

The description is as given in the proposal of the basonym (Lee et al., 2015), with the following addition. The genome size of the type strain is 3.86 Mbp. The DNA G + C content is 56.2 mol%.

Isolated from a saltern soil at Gomso (Korea).

The type strain is BH103^T^ = KACC 17609^T^ = NBRC 109914^T^ = NCAIM B 02528^T^.

Type strain genome sequence accession number: GCA_013415105.1.

Type strain 16S rRNA gene sequence accession number: KF963827.

### Description of *Vreelandella lutescens* comb. nov.

*Vreelandella lutescens* (lu.tes’cens. L. part. adj. *lutescens*, becoming muddy, related to the muddy color of the mature colony).

Basonym: *Halomonas lutescens* Wang *et al.* 2016.

The description is as given in the proposal of the basonym (Wang et al., 2016), with the following addition. The genome size of the type strain is 3.70 Mbp. The DNA G + C content is 56.0 mol%.

Isolated from a sediment sample from Qinghai Lake (China).

The type strain is Q1U^T^ = CGMCC 1.15122^T^ = KCTC 42517^T^.

Type strain genome sequence accession number: GCA_014640815.1.

Type strain 16S rRNA gene sequence accession number: KP259554.

### Description of *Vreelandella nigrificans* comb. nov.

*Vreelandella nigrificans* (nig.rif’i.cans. L. part. adj. *nigrificans*, making black).

Basonym: *Halomonas nigrificans* Oguntoyinbo *et al.* 2018.

The description is as given in the proposal of the basonym (Oguntoyinbo et al., 2018), with the following addition. The genome size of the type strain is 4.93 Mbp. The DNA G + C content is 52.8 mol%.

Isolated from cheese (Germany).

The type strain is MBT G8648^T^ = DSM 105749^T^ = LMG 29097^T^.

Type strain genome sequence accession number: GCA_002374315.1.

Type strain 16S rRNA gene sequence accession number: MG030686.

### Description of *Vreelandella malpeensis* comb. nov.

*Vreelandella malpeensis* (mal.pe.en’sis. N.L. fem. adj. *malpeensis*, of or belonging to Malpe, a coastal town in Udupi City, Karnataka, India).

Basonym: *Halomonas malpeensis* Kämpfer *et al.* 2018.

The description is as given in the proposal of the basonym (Kämpfer et al., 2018), with the following addition. The genome size of the type strain is 3.61 Mbp. The DNA G + C content is 63.8 mol%.

Isolated from the rhizosphere of sand dune coastal plant, Coast of Malpe (India).

The type strain is YU-PRIM-29^T^ = CCM 8737^T^ = LMG 28855^T^.

Type strain Genome sequence accession number: GCA_020622355.1.

Type strain 16S rRNA gene sequence accession number: JQ730736.

### Description of *Vreelandella piezotolerans* comb. nov.

*Vreelandella piezotolerans* (pie.zo.to’le.rans. Gr. v. *piezô*, to press; L. pres. part. *tolerans*, tolerating; N.L. part. adj. *piezotolerans*, pressure-tolerating).

Basonym: *Halomonas piezotolerans* Yan *et al.* 2020.

The description is as given in the proposal of the basonym (Yan et al., 2020), with the following addition. The genome size of the type strain is 3.95 Mbp. The DNA G + C content is 57.9 mol%.

Isolated from a deep-sea sediment sample of the New Britain Trench.

The type strain is NBT06E8^T^ = KCTC 72680^T^ = MCCC 1K04228^T^.

Type strain genome sequence accession numbers: GCA_012427705.1 and GCA_009660035.1.

Type strain 16S rRNA gene sequence accession number: MN435603.

### Description of *Vreelandella rituensis* comb. nov.

*Vreelandella rituensis* (ri.tu.en’sis. N.L. fem. adj. *rituensis*, pertaining to Ritu, Tibet, where the type strain was isolated).

Basonym: *Halomonas rituensis* Gao *et al.* 2020.

The description is as given in the proposal of the basonym (Gao et al., 2020), with the following addition. The genome size of the type strain is 4.47 Mbp. The DNA G + C content is 57.2 mol%.

Isolated from a salt marsh sediment of a saline lake, Dongqian Lake, (33°31′ 51.06″N 80°14′13.64″E), Tibetan Plateau (China).

The type strain is TQ8S^T^ = CICC 24572^T^ = KCTC 62530^T^.

Type strain genome sequence accession number: GCA_003336665.1.

Type strain 16S rRNA gene sequence accession number: MH071181.

### Description of *Vreelandella zhuhanensis* comb. nov.

*Vreelandella zhuhanensis* (zhu.han.en’sis. N.L. fem. adj. *zhuhanensis*, pertaining to Zhuhan marsh on the Tibetan Plateau, where the type strain was isolated).

Basonym: *Halomonas zhuhanensis* Gao *et al.* 2020.

The description is as given in the proposal of the basonym (Gao et al., 2020), with the following addition. The genome size of the type strain is 3.25 Mbp. The DNA G + C content is 57.1 mol%.

Isolated from a saline lake, Zhuhan Lake, (33°32′50.89″N 80°09′38.51″E), Tibetan Plateau (China).

The type strain is ZH2S^T^ = CICC 24505^T^ = KCTC 62531^T^.

Type strain genome sequence accession number: GCA_009793355.1.

Type strain 16S rRNA gene sequence accession number: MH071182.

### Description of *Vreelandella azerica* comb. nov.

*Vreelandella azerica* (a.ze’ri.ca. N.L. fem. adj. *azerica*, pertaining to Azerbaijan, where the Urmia Lake is located and the type strain was isolated).

Basonym: *Halomonas azerica* Wenting *et al.* 2021.

The description is as given in the proposal of the basonym (Wenting et al., 2021), with the following addition. The genome size of the type strain is 3.42 Mbp. The DNA G + C content is 55.4 mol%.

Isolated from Urmia Lake (Iran).

The type strain is TBZ9^T^ = KACC 21783^T^ = LMG 25775^T^.

Type strain genome sequence accession number: GCA_013112225.1.

Type strain 16S rRNA gene sequence accession number: MN900573.

### Description of *Vreelandella profundi* comb. nov.

*Vreelandella profundi* (pro.fun’di. L. gen. n. *profundi*, of the depth of the sea).

Basonym: *Halomonas profundi* Wang *et al.* 2022.

The description is as given in the proposal of the basonym (Wang et al., 2022), with the following addition. The genome size of the type strain is 3.60 Mbp. The DNA G + C content is 54.0 mol%.

Isolated from the deep-sea sediment of the Mariana Trench (11.12°N, 142.32°E).

The type strain is MT13^T^ = KCTC 82923^T^ = MCCC 1K06389^T^.

Type strain genome sequence accession number: GCA_019504685.1.

Type strain 16S rRNA gene sequence accession number: MZ411491.

### Description of *Vreelandella populi* comb. nov.

*Vreelandella populi* (po’pu.li. L. gen. n. *populi*, of the genus *Populus*, referring to *Populus euphratica*).

Basonym: *Halomonas populi* Xu *et al.* 2021.

The description is as given in the proposal of the basonym (Xu et al., 2021), with the following addition. The genome size of the type strain is 3.80 Mbp. The DNA G + C content is 55.0 mol%.

Isolated from *Populus euphratica* in Ebinur Lake Wetland Nature Reserve (China).

The type strain is MC^T^ = MCCC 1K03942^T^ = JCM 33545^T^.

Type strain genome sequence accession number: GCA_003989825.1.

Type strain 16S rRNA gene sequence accession number: MK045667.

### Description of *Vreelandella glaciei* sp. nov.

*Vreelandella glaciei* (gla.ci.e’i. L. gen. n. *glaciei*, meaning of the cold).

The description is as given in the original proposal of “*Halomonas glaciei*” (Reddy et al., 2003), with the following addition. The genome size of the type strain is 4.96 Mbp. The DNA G + C content is 54.4 mol%.

Isolated from the fast ice of Adelie Land, Antarctica.

The type strain is DD 39^T^ = CGMCC 1.7263^T^ = JCM 11692^T^ = MTCC 4321^T^.

Type strain genome sequence accession number: GCA_013415125.1.

Type strain 16S rRNA gene sequence accession number: AJ431369.

### Description of *Vreelandella zhaodongensis* sp. nov.

*Vreelandella zhaodongensis* (zhao.dong.en’sis. N.L. fem. adj. *zhaodongensis*, pertaining to Zhaodong City, North East of China, where the strain was isolated).

The description is as given in the original proposal of “*Halomonas zhaodongensis*” (Jiang et al., 2013), with the following addition. The genome size of the type strain is 3.72 Mbp. The DNA G + C content is 53.0 mol%.

Isolated from saline-alkaline soils in Zhaodong (China).

The type strain is NEAU-ST10-25^T^ = CGMCC 1.12286^T^ = DSM 25869^T^.

Type strain genome sequence accession number: GCA_013415115.1.

Type strain 16S rRNA gene sequence accession number: JQ762286.

### Description of *Vreelandella lionensis* sp. nov.

*Vreelandella lionensis* (li.on.en’sis. N.L. fem. adj. *lionensis*, of or belonging to Golfe du Lion [Gulf of Lions], in reference to the origin of the type strain).

The description is as given in the original proposal of “*Halomonas lionensis*” (Gaboyer et al., 2014), with the following addition. The genome size of the type strain is 3.65 Mbp. The DNA G + C content is 55.9 mol%.

Isolated from the Mediterranean Sea sediment, Gulf of Lions (France).

The type strain is RHS90^T^ = CIP 110370^T^ = DSM 25632^T^ = UBOCC 3186^T^.

Type strain genome sequence accession number: GCA_002087295.1.

Type strain 16S rRNA gene sequence accession number: HE661586.

### Description of *Vreelandella massiliensis* sp. nov.

*Vreelandella massiliensis* (mas.si.li.en’sis. L. fem. adj. *massiliensis*, of Massilia, the old Roman name for Marseille, where the strain was isolated).

The description is as given in the original proposal of “*Halomonas massiliensis*” (Seck et al., 2016), with the following addition. The genome size of the type strain is 3.44 Mbp. The DNA G + C content is 58.4 mol%.

Isolated from the human gut (France).

The type strain is Marseille-P2426^T^ = CSUR P2426^T^ = DSM 103116^T^.

Type strain genome sequence accession number: GCA_900155385.1.

Type strain 16S rRNA gene sequence accession number: LT223576.

### Description of *Vreelandella maris* sp. nov.

*Vreelandella maris* (ma’ris. L. gen. n. *maris*, of the sea).

The description is as given in the original proposal of “*Halomonas maris*” (Qiu et al., 2021a), with the following addition. The genome size of the type strain is 4.52 Mbp. The DNA G + C content is 54.4 mol%.

Isolated from the deep-sea sediment in the Southwest Indian Ocean (China).

The type strain is QX-1^T^ = KCTC 82198^T^ = MCCC 1A17875^T^ = NBRC 114670^T^.

Type strain genome sequence accession number: GCA_013371085.1.

Type strain 16S rRNA gene sequence accession number: MT372903.

### Description of *Vreelandella sedimenti* sp. nov.

*Vreelandella sedimenti* (se.di.men’ti. L. gen. n. *sedimenti*, of sediment, referring to the sediment of the Southwest Indian Ocean, where the type strain was isolated).

The description is as given in the original proposal of “*Halomonas sedimenti*” (Qiu et al., 2021b), with the following addition. The genome size of the type strain is 5.06 Mbp. The DNA G + C content is 54.3 mol%.

Isolated from the deep-sea sediment in the Southwest Indian Ocean (China).

The type strain is QX-2^T^ = KCTC 82199^T^ = MCCC 1A17876^T^.

Type strain genome sequence accession number: GCA_013416325.1.

Type strain 16S rRNA gene sequence accession number: MT372904.

### Description of *Bisbaumannia* gen. nov.

*Bisbaumannia* (Bis.bau.mann’i.a. L. adv. *bis*, twice; N.L. fem. n. *Bisbaumannia*, referring to both microbiologist Linda Baumann and Paul Baumann, who first studied these microorganisms).

Cells are Gram-staining-negative straight rods, 0.8–1.1 × 1.5–3.0 μm in size, aerobic, and motile by means of peritrichous flagella. Endospores are not formed. Oxidase-positive. Colonies are convex with entire edges and cream-colored. Na^+^ is required for growth. Mesophilic. Chemo-organotrophic. Nitrate reduction is negative. The major respiratory quinone is Q9. The major fatty acids are C_18:1_*ω*7*c*, C_19:0_ cyclo *ω*8*c*, C_16:0_, and C_16:1_*ω*7*c*. The major polar lipids are phosphatidylglycerol, diphosphatidylglycerol, and phosphatidylethanolamine.

The DNA G + C content is 67.2 mol%.

The genus *Bisbaumannia* belongs to the family *Halomonadaceae*. The type species is *Bisbaumannia pacifica.*

### Description of *Bisbaumannia pacifica* comb. nov.

*Bisbaumannia pacifica* (pa.ci’fi.ca. L. fem. adj. *pacifica*, peaceful, pertaining to the Pacific Ocean).

Basonym: *Alcaligenes pacificus* corrig. Baumann *et al.* 1972 (Approved Lists 1980).

Homotypic synomyms: *Halomonas pacifica* (Baumann *et al.* 1972) Dobson and Franzmann 1996; *Deleya pacifica* (Baumann *et al.* 1972) Baumann *et al.* 1983.

The description is as given in the original proposal of the basonym (Baumann et al., 1972), with the following addition. The genome size of the type strain is 3.85 Mbp. The DNA G + C content is 67.2 mol%.

Isolated from the seawater off the coast of Oahu (Hawaii, United States), Pacific Ocean.

The type strain is 62^T^ = ATCC 27122^T^ = CIP 103200^T^ = DSM 4742^T^ = JCM 20633^T^ = LMG 3446^T^ = NBRC 102220^T^ = NCIMB 1977^T^.

Type strain genome sequence accession number: GCA_007989625.1.

Type strain 16S rRNA gene sequence accession number: AB681734.

### Description of *Billgrantia* gen. nov.

*Billgrantia* (Bill.grant’i.a. N.L. fem. n. *Billgrantia*, named after the microbiologist William [Bill] D. Grant for his great contribution to the study of halophilic microorganisms).

Cells are Gram-staining-negative rods, 0.3–1.1 × 0.8–6.0 μm in size, aerobic or facultatively anaerobic, and mostly motile. Endospores are not formed. Catalase and oxidase are positive for most of the strains. Colonies are brown, cream, light beige, pinkish white, white, or yellow pigmented. Slightly to moderately halophilic, growing at 0–26% (w/v) NaCl, with optimal growth at 1–15% (w/v) NaCl. Mesophilic, thriving at 4–55°C, showing optimal growth at 25–42°C. Alkaliphilic or alkalitolerant, growing at pH values in the range of 5.0–12.0, with optimal growth at pH 7.0–10.0. Chemo-organotrophic. Nitrate reduction is mostly positive. The major respiratory quinones are Q9 and Q8. The major fatty acids are C_18:1_*ω*6*c*/C_18:1_*ω*7*c*, C_16:0_, C_16:1_*ω*6*c*/C_16:1_*ω*7*c*/iso-C_15:0_ 2-OH, C_19:0_ cyclo *ω*8*c*, C_12:0_ 3-OH, C_16:1_*ω*9c, and C_17:1_*ω*9c. The major polar lipids are diphosphatidylglycerol, phosphatidylglycerol, and phosphatidylethanolamine.

The DNA G + C content ranges between 62.1 and 67.5 mol%.

The genus *Billgrantia* belongs to the family *Halomonadaceae*. The type species is *Billgrantia desiderata*.

### Description of *Billgrantia desiderata* comb. nov.

*Billgrantia desiderata* (de.si.de.ra’ta. L. fem. adj. *desiderata*, wished for, the strain wished for).

Basonym: *Halomonas desiderata* Berendes *et al.* 1997.

The description is as given in the original proposal of the basonym (Berendes et al., 1996), with the following addition. The DNA G + C content is 64.7–64.9 mol%.

The type strain is FB2^T^ = CIP 105505^T^ = DSM 9502^T^ = LMG 19548^T^. The genome size of the type strain is 4.89 Mbp, and its DNA G + C content is 64.7 mol%. Isolated from a municipal sewage treatment plant in Göttingen (Germany).

Type strain genome sequence accession number: GCA_011742915.1.

Type strain 16S rRNA gene sequence accession number: X92417.

### Description of *Billgrantia campisalis* comb. nov.

*Billgrantia campisalis* (cam.pi.sa’lis. L. masc. n. *campus*, field, plain; L. masc. n. *sal*, salt; N.L. gen. n. *campisalis*, of the plain of salt, of the salt plain).

Basonym: *Halomonas campisalis* Mormile *et al.* 2000.

The description is as given in the original proposal of the basonym (Mormile et al., 1999), with the following addition. The genome size of the type strain is 4.27 Mbp. The DNA G + C content is 66.3 mol%.

Isolated from a soil sample collected from a salt flat south of Alkali Lake, Washington State (USA).

The type strain is 4A^T^ = ATCC 700597^T^ = CIP 106639^T^ = DSM 15413^T^.

Type strain genome sequence accession numbers: GCA_031451595.1 and GCA_022341425.1.

Type strain 16S rRNA gene sequence accession number: AF054286.

### Description of *Billgrantia gudaonensis* comb. nov.

*Billgrantia gudaonensis* (gu.dao.nen’sis. N.L. fem. adj. *gudaonensis*, pertaining to Gudao, in the Shengli oilfield, PR China, where the type strain was isolated).

Basonym: *Halomonas gudaonensis* Wang *et al.* 2007.

The description is as given in the original proposal of the basonym (Wang et al., 2007), with the following addition. The genome size of the type strain is 4.17 Mbp. The DNA G + C content is 64.9 mol%.

Isolated from saline soil contaminated by crude oil, Gudao (China).

The type strain is SL014B-69^T^ = CGMCC 1.6133^T^ = DSM 23417^T^ = LMG 23610^T^.

Type strain genome sequence accession number: GCA_900100195.1.

Type strain 16S rRNA gene sequence accession number: DQ421808.

### Description of *Billgrantia kenyensis* comb. nov.

*Billgrantia kenyensis* (ke.ny.en’sis. N.L. fem. adj. *kenyensis*, Kenyan, of Kenya, the region of isolation).

Basonym: *Halomonas kenyensis* Boltyanskaya *et al.* 2008.

The description is as given in the original proposal of the basonym (Boltyanskaya et al., 2007), with the following addition. The genome size of the type strain is 4.42 Mbp. The DNA G + C content is 63.8 mol%.

Isolated from sediments from soda lakes (Kenya).

The type strain is AIR-2^T^ = DSM 17331^T^ = VKM B-2354^T^.

Type strain genome sequence accession number: GCA_013697085.1.

Type strain 16S rRNA gene sequence accession number: AY962237.

### Description of *Billgrantia saliphila* comb. nov.

*Billgrantia saliphila* (sa.li’phi.la. L. masc. n. *sal*, salt; Gr. masc. adj. *philos*, loving; N.L. fem. adj. *saliphila*, salt-loving).

Basonym: *Halomonas saliphila* Gan *et al.* 2018.

The description is as given in the original proposal of the basonym (Gan et al., 2018), with the following addition. The genome size of the type strain is 4.34 Mbp. The DNA G + C content is 64.1 mol%.

Isolated from saline soil (China).

The type strain is LCB169^T^ = CGMCC 1.15818^T^ = KCTC 52618^T^.

Type strain genome sequence accession number: GCA_002930105.1.

Type strain 16S rRNA gene sequence accession number: KX008964.

### Description of *Billgrantia endophytica* comb. nov.

*Billgrantia endophytica* (en.do.phy’ti.ca. Gr. pref. *endo-*, within; Gr. neut. n. *phyton*, plant; L. fem. adj. suff. *-ica*, adjectival suffix used with the sense of belonging to; N.L. fem. adj. *endophytica*, within plant, pertaining to the endophytic nature of the strain and its isolation from internal plant tissues).

Basonym: *Halomonas endophytica* Chen *et al.* 2018.

The description is as given in the original proposal of the basonym (Chen et al., 2018), with the following addition. The genome size of the type strain is 4.98 Mbp. The DNA G + C content is 62.1 mol%.

Isolated from liquid in the stems of *Populus euphratica* in Xinjiang (China).

The type strain is MC28^T^ = KCTC 52999^T^ = MCCC 1K03343^T^.

Type strain genome sequence accession number: GCA_002879615.1.

Type strain 16S rRNA gene sequence accession number: MF850257.

### Description of *Billgrantia montanilacus* comb. nov.

*Billgrantia montanilacus* (mon.ta.ni.la’cus. L. masc. adj. *montanus*, a mountain; L. masc. n. *lacus*, lake; N.L. gen. n. *montanilacus*, of a mountain lake).

Basonym: *Halomonas montanilacus* Lu *et al.* 2020.

The description is as given in the original proposal of the basonym (Lu et al., 2020), with the following addition. The genome size of the type strain is 4.79 Mbp. The DNA G + C content is 62.9 mol%.

Isolated from hypersaline Lake Pengyanco on the Tibetan Plateau (China).

The type strain is PYC7W^T^ = CICC 24506^T^ = KCTC 62529^T^.

Type strain genome sequence accession number: GCA_003336675.1.

Type strain 16S rRNA gene sequence accession number: MH071180.

### Description of *Billgrantia lactosivorans* comb. nov.

*Billgrantia lactosivorans* (lac.to.si.vo’rans. L. neut. adj. *lactosum*, lactose; L. pres. part. *vorans*, eating; N.L. part. adj. *lactosivorans*, eating lactose).

Basonym: *Halomonas lactosivorans* Ming *et al.* 2020.

The description is as given in the original proposal of the basonym (Ming et al., 2020), with the following addition. The genome size of the type strain is 4.36 Mbp. The DNA G + C content is 66.7 mol%.

Isolated from salt-lake sediment in Shanxi Province (China).

The type strain is CFH 90008^T^ = DSM 103220^T^ = KCTC 52281^T^.

Type strain genome sequence accession number: GCA_003254665.1.

Type strain 16S rRNA gene sequence accession number: KY039330.

### Description of *Billgrantia pellis* comb. nov.

*Billgrantia pellis* (pel’lis. L. gen. n. *pellis*, of a hide, indicating the source of the type strain).

Basonym: *Halomonas pellis* Li *et al.* 2020.

The description is as given in the original proposal of the basonym (Li et al., 2020), with the following addition. The genome size of the type strain is 4.35 Mbp. The DNA G + C content is 63.6 mol%.

Isolated from wet salted hides (China).

The type strain is L5^T^ = CGMCC 1.17335^T^ = KCTC 72573^T^.

Type strain genome sequence accession number: GCA_008297955.1.

Type strain 16S rRNA gene sequence accession number: MN099429.

### Description of *Billgrantia azerbaijanica* comb. nov.

*Billgrantia azerbaijanica* (a.zer.bai.ja’ni.ca. N.L. fem. adj. *azerbaijanica*, pertaining to Azerbaijan, a region in the north-west of Iran, where Urmia Lake is located and from which the type strain was isolated).

Basonym: *Halomonas azerbaijanica* Kazemi *et al.* 2021.

The description is as given in the original proposal of the basonym (Kazemi et al., 2021), with the following addition. The genome size of the type strain is 4.58 Mbp. The DNA G + C content is 67.5 mol%.

Isolated from water from Urmia Lake (Iran).

The type strain is TBZ202^T^ = CECT 9693^T^ = KCTC 62817^T^.

Type strain genome sequence accession number: GCA_004551485.1.

Type strain 16S rRNA gene sequence accession number: MK138622.

### Description of *Billgrantia diversa* comb. nov.

*Billgrantia diversa* (di.ver’sa. L. fem. part. adj. *diversa*, different, distinct).

Basonym: *Halomonas diversa* Wang *et al.* 2021.

The description is as given in the original proposal of the basonym (Wang et al., 2021), with the following addition. The genome size of the type strain is 4.49 Mbp. The DNA G + C content is 62.9 mol%.

Isolated from the deep-sea sediment of the Pacific Ocean.

The type strain is D167-6-1^T^ = KCTC 72441^T^ = MCCC 1A13316^T^.

Type strain genome sequence accession number: GCA_014931605.1.

Type strain 16S rRNA gene sequence accession number: MW172430.

### Description of *Billgrantia bachuensis* comb. nov.

*Billgrantia bachuensis* (ba.chu.en’sis. N.L. fem. adj. *bachuensis*, pertaining to Bachu, north-western China, where the strain was isolated).

Basonym: *Halomonas bachuensis* Xiao *et al.* 2021.

The description is as given in the original proposal of the basonym (Xiao et al., 2021), with the following addition. The genome size of the type strain is 4.70 Mbp. The DNA G + C content is 63.6 mol%.

Isolated from Gobi soil, Bachu (China).

The type strain is DX6^T^ = CCTCC AB 2020094^T^ = KCTC 82196^T^.

Type strain genome sequence accession number: GCA_011742165.1.

Type strain 16S rRNA gene sequence accession number: MT180568.

### Description of *Billgrantia antri* comb. nov.

*Billgrantia antri* (an’tri. L. gen. n. *antri*, of a cave, referring to the location of the isolate).

Basonym: *Halomonas antri* So *et al.* 2022.

The description is as given in the original proposal of the basonym (So et al., 2022), with the following addition. Cells are Gram-stain-negative rods, 0.6–0.9 × 1.6–2.4 μm in size, strictly aerobic or facultatively anaerobic, and motile by means of one polar flagellum or peritrichous flagella. Catalase and oxidase activities are variable. Slightly to moderately halophilic, growing at 0–20% (w/v) NaCl, with optimal growth at 1–8% (w/v) NaCl. Mesophilic, thriving at 4–55°C, showing optimal growth at 25–40°C. Alkalitolerant, growing at pH values in the range of 6.0–10.0, with optimal growth at pH 7.0–8.0. Voges–Proskauer test is variable. Starch is hydrolyzed, but Tween 40 and Tween 60 are not. Hydrolysis of DNA and urea is variable. Esterase lipase (C8), lipase (C14), cystine arylamidase, α-glucosidase, and tryptophan deaminase activities are variable. Utilization of N-acetyl-glucosamine, potassium gluconate, adipate, and citrate is variable. The major respiratory quinone is Q9. The major fatty acids are C_18:1_*ω*6*c*/C_18:1_*ω*7*c*, C_16:0_, C_16:1_*ω*6*c*/C_16:1_*ω*7*c*, C_19:0_ cyclo *ω*8*c*, and C_12:0_ 3-OH. The major polar lipids are diphosphatidylglycerol, phosphatidylglycerol, and phosphatidylethanolamine.

The DNA G + C content is 64.2–64.3 mol%.

The type strain is Y3S6^T^ = KACC 21536^T^ = NBRC 114315^T^ = TBRC 15164^T^. The genome size of the type strain is 4.39 Mbp, and its DNA G + C content is 64.3 mol%. Isolated from surface seawater, Busan (Korea).

Type strain genome sequence accession number: GCA_019430905.1.

Type strain 16S rRNA gene sequence accession number: MN625868.

### Description of *Billgrantia chromatireducens* sp. nov.

*Billgrantia chromatireducens* (chro.ma.ti.re.du’cens. N.L. masc. n. *chromas*, chromate; L. pres. part. *reducens*, converting to a different state; N.L. part. adj. *chromatireducens*, reducing chromate).

The description is as given in the original proposal of “*Halomonas chromatireducens*” (Shapovalova et al., 2009), with the following addition. The genome size of the type strain is 3.97 Mbp. The DNA G + C content is 62.8 mol%.

Isolated from soda salt marshes, Altai (Russia).

The type strain is AGD 8-3^T^ = NCCB 100225^T^ = VKM B-2497^T^.

Type strain genome sequence accession number: GCA_001545155.1.

Type strain 16S rRNA gene sequence accession number: EU447163.

### Description of *Billgrantia aerodenitrificans* sp. nov.

*Billgrantia aerodenitrificans* (a.e.ro.de.ni.tri’fi.cans. Gr. masc. n. *aêr*, air; N.L. inf. v. *denitrificare*, to denitrify; N.L. part. adj. *aerodenitrificans*, denitrifying with or in air).

The description is as given in the original proposal of “*Halomonas aerodenitrificans*” (Wang and Shao, 2021), with the following addition. The genome size of the type strain is 5.08 Mbp. The DNA G + C content is 64.0 mol%.

Isolated from coastal water, 5 m depth, from the Taiwan Strait (China).

The type strain is CYD-9^T^ = KCTC 72088^T^ = MCCC 1A11058^T^.

Type strain genome sequence accession number: GCA_021404405.1.

Type strain 16S rRNA gene sequence accession number: MW205680.

### Description of *Billgrantia ethanolica* sp. nov.

*Billgrantia ethanolica* (e.tha.no’li.ca. N.L. neut. n. *ethanol*, ethanol; L. fem. adj. suff. *-ica*, suffix used with various meanings; N.L. fem. adj. *ethanolica*, belonging to ethanol, in reference to the ability of the species to utilize ethanol as a substrate for growth).

The description is as given in the original proposal of “*Halomonas ethanolica*” (Wang and Shao, 2021), with the following addition. The genome size of the type strain is 4.57 Mbp. The DNA G + C content is 64.5 mol%.

Isolated from the sediment from shrimp culture pond, Zhangzhou (China).

The type strain is CYT3-1-1^T^ = KCTC 72090^T^ = MCCC 1A11081^T^.

Type strain genome sequence accession number: GCA_021404305.1.

Type strain 16S rRNA gene sequence accession number: MW205683.

### Description of *Billgrantia sulfidoxydans* sp. nov.

*Billgrantia sulfidoxydans* (sul.fid.o’xy.dans. N.L. neut. n. *sulfidum*, sulfide; N.L. pres. part. *oxydans*, oxidizing; N.L. part. adj. *sulfidoxydans*, oxidizing sulfides).

The description is as given in the original proposal of “*Halomonas sulfidoxydans*” (Wang and Shao, 2021), with the following addition. The genome size of the type strain is 4.49 Mbp. The DNA G + C content is 66.0 mol%.

Isolated from surface sediments in the coastal sea at Taiwan Strait (China).

The type strain is CYN-1-2^T^ = KCTC 72089^T^ = MCCC 1A11059^T^.

Type strain genome sequence accession number: GCA_017868775.1.

Type strain 16S rRNA gene sequence accession number: MW205681.

### Description of *Billgrantia tianxiuensis* sp. nov.

*Billgrantia tianxiuensis* (tian.xiu.en’sis. N.L. fem. adj. *tianxiuensis*, pertaining to the Tianxiu Hydrothermal Field, on the Northwest Indian Ridge, from where the type strain was isolated).

The description is as given in the original proposal of “*Halomonas tianxiuensis*” (Wang and Shao, 2021), with the following addition. The genome size of the type strain is 5.02 Mbp. The DNA G + C content is 63.9 mol%.

Isolated from sulfide from Tianxiu hydrothermal vents, 3,440 m depth, Northwest Indian Ocean.

The type strain is BC-M4-5^T^ = KCTC 72092^T^ = MCCC 1A14433^T^.

Type strain genome sequence accession number: GCA_009834345.1.

Type strain 16S rRNA gene sequence accession number: MW205685.

### Description of *Billgrantia zhangzhouensis* sp. nov.

*Billgrantia zhangzhouensis* (zhang.zhou.en’sis. N.L. fem. adj. *zhangzhouensis*, of or pertaining to Zhangzhou, a city in Fujian, China, where the type strain was isolated).

The description is as given in the original proposal of “*Halomonas zhangzhouensis*” (Wang and Shao, 2021), with the following addition. The genome size of the type strain is 4.41 Mbp. The DNA G + C content is 63.3 mol%.

Isolated from the sediments from a shrimp culture pond in Zhangzhou (China).

The type strain is CXT3-11^T^ = KCTC 72087^T^ = MCCC 1A11036^T^.

Type strain genome sequence accession number: GCA_021404465.1.

Type strain 16S rRNA gene sequence accession number: MW205678.

### Description of *Franzmannia* gen. nov.

*Franzmannia* (Franz.man’ni.a. N.L. fem. n. *Franzmannia*, in honor of Peter D. Franzmann, Australian microbiologist and polar researcher).

Cells are Gram-staining-negative rod-shaped or pleomorphic, 0.3–0.7 × 1.4–2.8 μm in size, aerobic, and motile or non-motile. Endospores are not formed. Catalase-variable and oxidase-positive. Colonies are cream, cream-pink, or light yellow pigmented. Moderately halophilic, growing at 0.5–20% (w/v) NaCl, with optimal growth at 2–10% (w/v) NaCl. Mesophilic, thriving at 10–45°C, showing optimal growth at 25–37°C. Alkalitolerant, growing at pH values in the range of 5.5–11.0, with optimal growth at pH 7.0–9.0. Chemo-organotrophic. Nitrate reduction is variable. The major respiratory quinone is Q9. The major fatty acids are C_18:1_*ω*6*c*/C_18:1_*ω*7*c*, C_16:0_, C_19:0_ cyclo *ω*8*c*, and C_16:1_*ω*6*c*/C_16:1_*ω*7*c*. The major polar lipids are phosphatidylglycerol, diphosphatidylglycerol, and phosphatidylethanolamine.

The DNA G + C content ranges between 63.8 and 64.5 mol%.

The genus *Franzmannia* belongs to the family *Halomonadaceae*. The type species is *Franzmannia pantelleriensis*.

### Description of *Franzmannia pantelleriensis* comb. nov.

*Franzmannia pantelleriensis* (pan.tel.le.ri.en’sis. L. fem. adj. *pantelleriensis*, pertaining to Pantelleria Island [the place of isolation] in the south of Sicily, Italy).

Basonym: *Halomonas pantelleriensis* corrig Romano *et al.* 1997.

The description is as given in the proposal of the basonym (Romano et al., 1996), with the following addition. The genome size of the type strain is 4.40 Mbp. The DNA G + C content is 63.9 mol%.

Isolated from the hard sand of Venere Lake, Pantelleria Island (Italy).

The type strain is AAP^T^ = ATCC 700273^T^ = CIP 105506^T^ = DSM 9661^T^ = LMG 19550^T^.

Type strain genome sequence accession number: GCA_900102875.1.

Type strain 16S rRNA gene sequence accession number: X93493.

### Description of *Franzmannia qiaohouensis* comb. nov.

*Franzmannia qiaohouensis* (qiao.hou.en’sis. N.L. fem. adj. *qiaohouensis*, pertaining to Qiaohou salt mine, south-west China, where the type strain was isolated).

Basonym: *Halomonas qiaohouensis* Wang *et al.* 2015.

The description is as given in the proposal of the basonym (Wang et al., 2014), with the following addition. The genome size of the type strain is 4.65 Mbp. The DNA G + C content is 64.5 mol%.

Isolated from the salt mine soil in southwest China.

The type strain is YIM QH88^T^ = ACCC 60021^T^ = CCTCC AB 2012965^T^ = DSM 26770^T^.

Type strain genome sequence accession number: GCA_031451695.1.

Type strain 16S rRNA gene sequence accession number: KC237714.

### Description of *Franzmannia salipaludis* sp. nov.

*Franzmannia salipaludis* (sa.li.pa.lu’dis. L. masc. n. *sal*, salt; L. fem. n. *palus*, swamp, marsh; N.L. gen. n. *salipaludis*, of a salt marsh).

The description is as given in the original proposal of “*Halomonas salipaludis*” (Xing et al., 2021), with the following addition. The genome size of the type strain is 5.48 Mbp. The DNA G + C content is 63.8 mol%.

Isolated from the saline-alkali wetland soil (China).

The type strain is WRN001^T^ = ACCC 19974^T^ = KCTC 52853^T^.

Type strain genome sequence accession number: GCA_002286975.1.

Type strain 16S rRNA gene sequence accession number: MF782428.

### Description of *Litchfieldella* gen. nov.

*Litchfieldella* (Litch.field.el’la. N.L. fem. n. *Litchfieldella*, named after Dr. Carol D. Litchfield [1936–2012], in recognition of her many contributions to the study of halophilic microorganisms).

Cells are Gram-staining-negative rods, 0.4–1.0 × 1.2–4.4 μm in size, aerobic, and motile or non-motile. Endospores are not formed. Catalase-positive and oxidase-variable. Colonies are cream, yellow, brown-orange, or creamy-white pigmented. Moderately halophilic, growing at 0–23% (w/v) NaCl, with optimal growth at 5–13% (w/v) NaCl. Mesophilic, thriving at 15–50°C, showing optimal growth at 32–37°C. Alkalitolerant, growing at pH values in the range of 5.0–10.0, with optimal growth at pH 6.0–9.0. Chemo-organotrophic. Nitrate reduction is variable. The major respiratory quinone is Q9. The major fatty acids are C_18:1_*ω*7*c*, C_16:0_, C_16:1_*ω*7*c*/iso-C_15:0_ 2-OH, and C_19:0_ cyclo *ω*8*c*.

The DNA G + C content ranges between 58.5 and 62.2 mol%.

The genus *Litchfieldella* belongs to the family *Halomonadaceae*. The type species is *Litchfieldella anticariensis*.

### Description of *Litchfieldella anticariensis* comb. nov.

*Litchfieldella anticariensis* (an.ti.ca.ri.en’sis. L. fem. adj. *anticariensis*, pertaining to Antequera, originally the Roman city of Anticaria, in the province of Málaga, southern Spain, where the strains were isolated).

Basonym: *Halomonas anticariensis* Martínez-Cánovas *et al.* 2004.

The description is as given in the proposal of the basonym (Martínez-Cánovas et al., 2004), with the following addition. The genome size of the type strain is 5.07 Mbp. The DNA G + C content is 58.5 mol%.

Isolated from a soil sample from Fuente de Piedra, a saline-wetland wildfowl reserve in Málaga (Spain).

The type strain is FP35^T^ = CECT 5854^T^ = CIP 108499^T^ = DSM 16096^T^ = LMG 22089^T^.

Type strain genome sequence accession numbers: GCA_000409775.1 and GCA_000428505.1.

Type strain 16S rRNA gene sequence accession number: AY489405.

### Description of *Litchfieldella xinjiangensis* comb. nov.

*Litchfieldella xinjiangensis* (xin.ji.ang.en’sis. N.L. fem. adj. *xinjiangensis*, pertaining to Xinjiang, a region of China, from where the type strain was isolated).

Basonym: *Halomonas xinjiangensis* Guan *et al.* 2010.

The description is as given previously (Guan et al., 2010; Navarro-Torre et al., 2020), with the following addition. The genome size of the type strain is 3.79 Mbp. The DNA G + C content is 60.7 mol%.

Isolated from a soil sample from Lop Nur salt lake (4° 23′ N 9° 18′ E, 778 m altitude) in Xinjiang Province, north-west China.

The type strain is TRM 0175^T^ = CCTCC AB 208329^T^ = KCTC 22608^T^.

Type strain genome sequence accession number: GCA_000759345.1.

Type strain 16S rRNA gene sequence accession number: EU822512.

### Description of *Litchfieldella rifensis* comb. nov.

*Litchfieldella rifensis* (ri.fen’sis. N.L. fem. adj. *rifensis*, pertaining to the Rif Mountains in northern Morocco, where the strain was isolated).

Basonym: *Halomonas rifensis* Amjres *et al.* 2011.

The description is as given in the proposal of the basonym (Amjres et al., 2011), with the following addition. The genome size of the type strain is 4.83 Mbp. The DNA G + C content is 62.2 mol%.

Isolated from a solar saltern in the Rif Mountains (Morocco).

The type strain is HK31^T^ = CECT 7698^T^ = LMG 25695^T^.

Type strain genome sequence accession number: GCM10020179.

Type strain 16S rRNA gene sequence accession number: HM026177.

### Description of *Litchfieldella qijiaojingensis* comb. nov.

*Litchfieldella qijiaojingensis* (qi.jiao’jing.en’sis. N.L. fem. adj. *qijiaojingensis*, pertaining to Qijiaojing Lake, Xinjiang Province, north-west China, where the sample from which the type strain was isolated and was collected).

Basonym: *Halomonas qijiaojingensis* Chen *et al.* 2012.

The description is as given in the proposal of the basonym (Chen et al., 2011), with the following addition. The genome size of the type strain is 4.77 Mbp. The DNA G + C content is 60.8 mol%.

Isolated from the shore sediment from a salt lake, Qijiaojing Lake (43°23′01” N 91°36′11″ E), Xinjiang province (China).

The type strain is YIM 93003^T^ = CCTCC AB 208133^T^ = DSM 22403^T^ = KCTC 22228^T^.

Type strain genome sequence accession number: GCA_014651875.1.

Type strain 16S rRNA gene sequence accession number: HQ832735.

### Description of *Onishia* gen. nov.

*Onishia* (O.ni’shi.a. N.L. fem. n. *Onishia*, named after Dr. Hiroshi Ōnishi [Japan], in recognition of his many contributions to the study of halophilic microorganisms).

Cells are Gram-staining-negative rods, 0.6–1.0 × 1.8–3.2 μm in size, aerobic or facultatively anaerobic, and motile. Endospores are not formed. Catalase and oxidase are positive. Colonies are cream pigmented. Moderately halophilic, growing at 1–25% (w/v) NaCl, with optimal growth at 10–15% (w/v) NaCl. Mesophilic, thriving at 4–45°C, showing optimal growth at 22–35°C. Alkalitolerant, growing at pH values in the range of 4.0–10.0, with optimal growth at pH 7.5–8.0. Chemo-organotrophic. Nitrate reduction is positive. The major respiratory quinones are Q9 and, according to the genome sequence, also Q8. The major fatty acids are C_18:1_*ω*6*c*/C_18:1_*ω*7*c*, C_16:0_, C_16:1_*ω*6*c*/C_16:1_*ω*7*c*/iso-C_15:0_ 2-OH, C_19:0_ cyclo *ω*8*c*, and C_12:0_ 3-OH. According to the genome sequence, the major polar lipids are phosphatidylglycerol, phosphatidylethanolamine, and diphosphatidylglycerol.

The DNA G + C content ranges between 61.1 and 62.3 mol%.

The genus *Onishia* belongs to the family *Halomonadaceae*. The type species is *Onishia taeanensis*.

### Description of *Onishia taeanensis* comb. nov.

*Onishia taeanensis* (tae.an.en’sis. N.L. fem. adj. *taeanensis*, belonging to Taean, from where the organism was isolated).

Basonym: *Halomonas taeanensis* Lee *et al.* 2005.

The description is as given in the proposal of the basonym (Lee et al., 2005), with the following addition. The genome size of the type strain is 3.76 Mbp. The DNA G + C content is 62.3 mol%.

Isolated from the soil from a solar saltern (Korea).

The type strain is BH539^T^ = CIP 109003^T^ = DSM 16463^T^ = KCTC 12284^T^.

Type strain genome sequence accession number: GCA_900100755.1.

Type strain 16S rRNA gene sequence accession number: AY671975.

### Description of *Onishia niordana* comb. nov.

*Onishia niordana* (nior.di.a’na. N.L. fem. adj. *niordana*, pertaining to Njörd [Niord], Nordic god of marine coast).

Basonym: *Halomonas niordiana* Diéguez *et al.* 2020.

The description is as given in the proposal of the basonym (Diéguez et al., 2020), with the following addition. The genome size of the type strain is 3.68 Mbp. The DNA G + C content is 61.1 mol%.

Isolated from seawater (Norway).

The type strain is ATF 5.4^T^ = CECT 9779^T^ = LMG 31227^T^.

Type strain genome sequence accession number: GCA_004798965.1.

Type strain 16S rRNA gene sequence accession number: SDSD01000014.

### Emended description of the genus *Modicisalibacter* Ben Ali Gam *et al.* 2007

*Modicisalibacter* (Mo.di.ci.sa.li.bac’ter. L. masc. adj. *modicus*, moderate, limited; L. masc. n. *sal* [gen. *salis*], salt; N.L. masc. n. *bacter*, a rod; N.L. masc. n. *Modicisalibacter*, a moderately halophilic rod).

Cells are Gram-staining-negative rods or short rods, 0.1–1.0 × 0.2–4.0 μm in size, aerobic, and mostly motile. Endospores are not formed. Catalase-positive and oxidase-variable. Colonies are cream, orange, pale/light orange, or yellow pigmented or colorless. Moderately halophilic, growing at 0–27.5% (w/v) NaCl, with optimal growth at 2.5–10% (w/v) NaCl. Mesophilic, thriving at 4–45°C, showing optimal growth at 25–37°C. Alkalitolerant, growing at pH values in the range of 5.0–10.0, with optimal growth at pH 6.5–8.5. Chemo-organotrophic. Nitrate reduction is positive. The major respiratory quinones are Q9 and Q8. The major fatty acids are C_18:1_*ω*6*c*/C_18:1_*ω*7*c*, C_16:0_, C_16:1_*ω*7*c*/iso-C_15:0_ 2-OH, C_19:0_ cyclo *ω*8*c*, and C_12:0_ 3-OH. The major polar lipids are diphosphatidylglycerol, phosphatidylglycerol, and phosphatidylethanolamine.

The DNA G + C content ranges between 59.1 and 67.4 mol%.

The genus *Modicisalibacter* belongs to the family *Halomonadaceae*. The type species is *Modicisalibacter tunisiensis*.

### Description of *Modicisalibacter muralis* comb. nov.

*Modicisalibacter muralis* (mu.ra’lis. L. masc. adj. *muralis*, pertaining or belonging to walls).

Basonym: *Halomonas muralis* Heyrman *et al.* 2002.

The description is as given in the proposal of the basonym (Heyrman et al., 2002), with the following addition. The genome size of the type strain is 4.14 Mbp. The DNA G + C content is 61.9 mol%.

Isolated from microbial biofilms colonizing the walls and murals of the Saint-Catherine chapel, Castle Herberstein (Austria).

The type strain is R-5058^T^ = CIP 108825^T^ = DSM 14789^T^ = LMG 20969^T^.

Type strain genome sequence accession number: GCA_900102945.1.

Type strain 16S rRNA gene sequence accession number: AJ320530.

### Description of *Modicisalibacter luteus* comb. nov.

*Modicisalibacter luteus* (lu’te.us. L. masc. adj. *luteus*, orange-colored).

Basonym: *Halomonas lutea* Wang *et al.* 2008.

The description is as given in the proposal of the basonym (Wang et al., 2008), with the following addition. The genome size of the type strain is 4.53 Mbp. The DNA G + C content is 59.1 mol%.

Isolated from a salt lake, Xinjiang province (China).

The type strain is YIM 91125^T^ = CCTCC AB 206093^T^ = DSM 23508^T^ = KCTC 12847^T^.

Type strain genome sequence accession number: GCA_000378505.1.

Type strain 16S rRNA gene sequence accession number: EF674852.

### Description of *Modicisalibacter ilicicola* comb. nov.

*Modicisalibacter ilicicola* (i.li.ci’co.la. L. n. *ilici*, the Roman name of Elche, the city close to the solar salterns where the type strain was isolated; L. masc. n. suff. *-cola*, inhabitant, dweller; N.L. masc. n. *ilicicola*, inhabitant of Ilici).

Basonym: *Halomonas ilicicola* Arenas *et al.* 2009.

The description is as given in the proposal of the basonym (Arenas et al., 2009), with the following addition. The genome size of the type strain is 3.96 Mbp. The DNA G + C content is 63.2 mol%.

Isolated from saline water from a solar saltern, Santa Pola (38° 11′ 35” N 0° 35′ 45” W), Alicante (Spain).

The type strain is SP8^T^ = CCM 7522^T^ = CECT 7331^T^ = DSM 19980^T^.

Type strain genome sequence accession number: GCA_900128925.1.

Type strain 16S rRNA gene sequence accession number: EU218533.

### Description of *Modicisalibacter xianhensis* comb. nov.

*Modicisalibacter xianhensis* (xianh.en’sis. N.L. masc. adj. *xianhensis*, of or pertaining to Xianhe, Shandong Province, China, where the type strain was isolated).

Basonym: *Halomonas xianhensis* Zhao *et al.* 2012.

The description is as given in the proposal of the basonym (Zhao et al., 2012), with the following addition. The genome size of the type strain is 4.36 Mbp. The DNA G + C content is 61.3 mol%.

Isolated from saline soil contaminated with crude oil from Xianhe, Shandong Province (China).

The type strain is A-1^T^ = CGMCC 1.6848^T^ = JCM 14849^T^.

Type strain genome sequence accession number: GCA_900113605.1.

Type strain 16S rRNA gene sequence accession number: EF421176.

### Description of *Modicisalibacter zincidurans* comb. nov.

*Modicisalibacter zincidurans* (zin.ci.du’rans. N.L. neut. n. *zincum*, zinc; L. pres. part. *durans*, enduring, being insensible; N.L. part. adj. *zincidurans*, zinc tolerating).

Basonym: *Halomonas zincidurans* Xu *et al.* 2013.

The description is as given previously (Xu et al., 2013; Navarro-Torre et al., 2020), with the following addition. The genome size of the type strain is 3.55 Mbp. The DNA G + C content is 64.4 mol%.

Isolated from deep-sea sediment from the South Atlantic Ocean.

The type strain is B6^T^ = CGMCC 1.12450^T^ = JCM 18472^T^.

Type strain genome sequence accession number: GCA_000731955.1.

Type strain 16S rRNA gene sequence accession number: JQ781698.

### Description of *Modicisalibacter radicis* comb. nov.

*Modicisalibacter radicis* (ra’di.cis. L. gen. n. *radicis*, of a root).

Basonym: *Halomonas radicis* Navarro-Torre *et al.* 2020.

The description is as given in the proposal of the basonym (Navarro-Torre et al., 2020), with the following addition. The genome size of the type strain is 4.65 Mbp. The DNA G + C content is 64.9 mol%.

Isolated from *Arthrocnemum macrostachyum* growing in the Odiel marshes (Spain).

The type strain is EAR18^T^ = CECT 9077^T^ = LMG 29859^T^.

Type strain genome sequence accession number: GCA_900961225.1.

Type strain 16S rRNA gene sequence accession number: KU320882.

### Description of *Modicisalibacter coralii* sp. nov.

*Modicisalibacter coralii* (co.ra’li.i. L. gen. n. *coralii*, from the coral *Mussismilia braziliensis*).

The description is as given in the original proposal of “*Halomonas coralii*” (Vidal et al., 2019), with the following addition. The genome size of the type strain is 4.44 Mbp. The DNA G + C content is 66.3 mol%.

Isolated from *Mussismilia braziliensis* (Brazil).

The type strain is 362.1^T^ = CBAS 715^T^.

Type strain genome sequence accession number: GCA_004117855.1.

Type strain 16S rRNA gene sequence accession number: QWBW01000001.

## Data availability statement

The datasets presented in this study can be found in online repositories. The names of the repository/repositories and accession number(s) can be found in Supplementary material.

## Author contributions

RRH: Conceptualization, Formal analysis, Writing – original draft, Writing – review & editing. DRA: Conceptualization, Investigation, Writing – original draft, Writing – review & editing. CS-P: Conceptualization, Investigation, Writing – review & editing. MC: Formal analysis, Writing – review & editing. SW: Supervision, Writing – review & editing. PH: Supervision, Writing – review & editing. AV: Conceptualization, Writing – review & editing.

## Footnotes

^1^ https://lpsn.dsmz.de/text/largest-genera

^2^ https://www.ncbi.nlm.nih.gov/assembly

^3^ https://www.ncbi.nlm.nih.gov/sra

^4^ https://genome.jgi.doe.gov/portal

^5^ https://genomes.atcc.org

^6^ https://github.com/novigit/davinciCode/blob/master/perl

^7^ https://github.com/dparks1134/PhyloRank

^8^ https://github.com/SWittouck/tidygenomes

^9^ https://github.com/SWittouck/lacto_genera

^10^ https://lpsn.dsmz.de/family/hahellaceae

^11^ https://www.namesforlife.com/10.1601/tx.2487
