# Corrigendum: A long-awaited taxogenomic investigation of the family *Halomonadaceae*

**DOI:** 10.3389/fmicb.2025.1549745

**Published:** 2025-01-28

**Authors:** Rafael R. de la Haba, David R. Arahal, Cristina Sánchez-Porro, Maria Chuvochina, Stijn Wittouck, Philip Hugenholtz, Antonio Ventosa

**Affiliations:** ^1^Department of Microbiology and Parasitology, Faculty of Pharmacy, University of Sevilla, Sevilla, Spain; ^2^Department of Microbiology and Ecology, University of Valencia, Valencia, Spain; ^3^The University of Queensland, School of Chemistry and Molecular Biosciences, Australian Centre for Ecogenomics, St Lucia, QLD, Australia; ^4^Research Group Environmental Ecology and Applied Microbiology, Department of Bioscience Engineering, University of Antwerp, Antwerp, Belgium

**Keywords:** phylogenomics, signature genes, halophiles, taxonomic reclassification, genus delineation

In the published article, there was an error in the formation of the new combination *Vreelandella utahensis*, which should have been named *Vreelandella halophila* comb. nov. instead, using the earliest legitimate epithet of the species in application of Rule 41a (Oren et al., 2023).

A correction has been made to the Taxonomic conclusions section, specifically to the Description of *Vreelandella utahensis* comb. nov. This section previously stated:

“**Description of** ***Vreelandella utahensis*** **comb. nov**.

*Vreelandella utahensis* (u.ta.hen'sis. N.L. fem. adj. *utahensis*, referring to Utah).

Basonym: *Halomonas utahensis* Sorokin and Tindall, 2006.

The description is as given in the proposal of the basonym (Sorokin and Tindall, 2006), with the following addition. The genome size of the type strain is 3.73 Mbp. The DNA G + C content is 55.8 mol%.

Isolated from surface water from the North Arm of Great Salt Lake (United States).

The type strain is isolate III^T^ = ATCC 49240^T^ = CECT 5286^T^ = CIP 105504^T^ = DSM 3051^T^ = IAM 14440^T^ = JCM 21223^T^ = NBRC 102410^T^.

Type strain genome sequence accession number: GCA_007991175.1.

Type strain 16S rRNA gene sequence accession number: AJ306893.”

The corrected section appears below:

“**Description of** ***Vreelandella halophila*** **comb. nov**.

*Vreelandella halophila* (ha.lo'phi.la. Gr. masc. n. *hals* [gen. *halos*], salt; N.L. fem. adj. suff. *-phila*, friend, loving; from Gr. fem. adj. *philê*, loving; N.L. fem. adj. *halophila*, salt-loving).

Basonym: *Pseudomonas halophila* Fendrich 1989.

The description is as given in the proposal of the homotypic synonym *Halomonas utahensis* (Sorokin and Tindall, 2006), with the following addition. The genome size of the type strain is 3.73 Mbp. The DNA G + C content is 55.8 mol%.

Isolated from surface water from the North Arm of Great Salt Lake (United States).

The type strain is isolate III^T^ = ATCC 49240^T^ = CECT 5286^T^ = CIP 105504^T^ = DSM 3051^T^ = IAM 14440^T^ = JCM 21223^T^ = NBRC 102410^T^.

Type strain genome sequence accession number: GCA_007991175.1.

Type strain 16S rRNA gene sequence accession number: AJ306893.”

The authors apologize for this error and state that this does not change the scientific conclusions of the article in any way.
