# Supplementary material for: A long-awaited taxogenomic investigation of the family Halomonadaceae
Source: Front Microbiol. 2023 Nov 16;14:1293707. doi: 10.3389/fmicb.2023.1293707 (PMC10690426; doi:10.3389/fmicb.2023.1293707)
Supplement: Supplementary file 1 [file Data_Sheet_1.pdf]

Supplementary Material

A long-awaited taxogenomic investigation of the family *Halomonadaceae*

Rafael R. de la Haba[1*], David R. Arahal[2], Cristina Sánchez-Porro[1], Maria Chuvochina[3], Stijn Wittouck[4], Phil Hugenholtz[3], Antonio Ventosa[1*]

[1]Department of Microbiology and Parasitology, Faculty of Pharmacy, University of Sevilla, Sevilla, Spain
[2]Departament of Microbiology and Ecology, University of Valencia, Valencia, Spain
[3]Australian Centre for Ecogenomics, School of Chemistry and Molecular Biosciences, The University of Queensland, St Lucia, QLD, Australia
[4]Research Group Environmental Ecology and Applied Microbiology, Department of Bioscience Engineering, University of Antwerp, Antwerp, Belgium

*** Correspondence:**
Rafael R. de la Haba
rrh@us.es

Antonio Ventosa
ventosa@us.es

1 Supplementary Figures

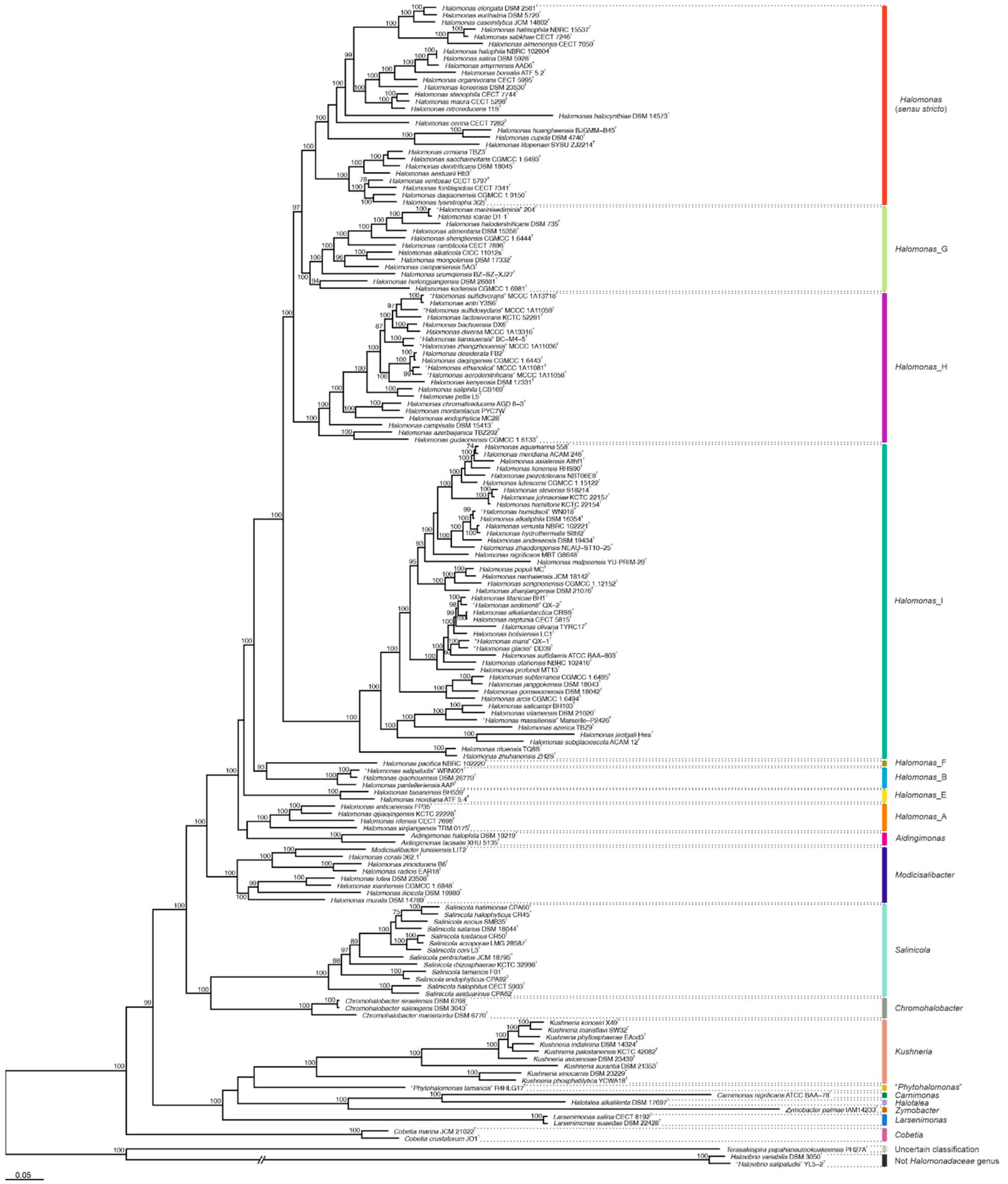

Supplementary Figure 1. Clade expanded maximum-likelihood phylogenomic tree based on the concatenation of the translated sequence of the 189 single-copy genes shared at least by 90% of the members of the family *Halomonadaceae* under study ("core90" set). Ultrafast bootstrap values ≥ 70% are shown above the branches. Bar, 0.05 changes per nucleotide position.

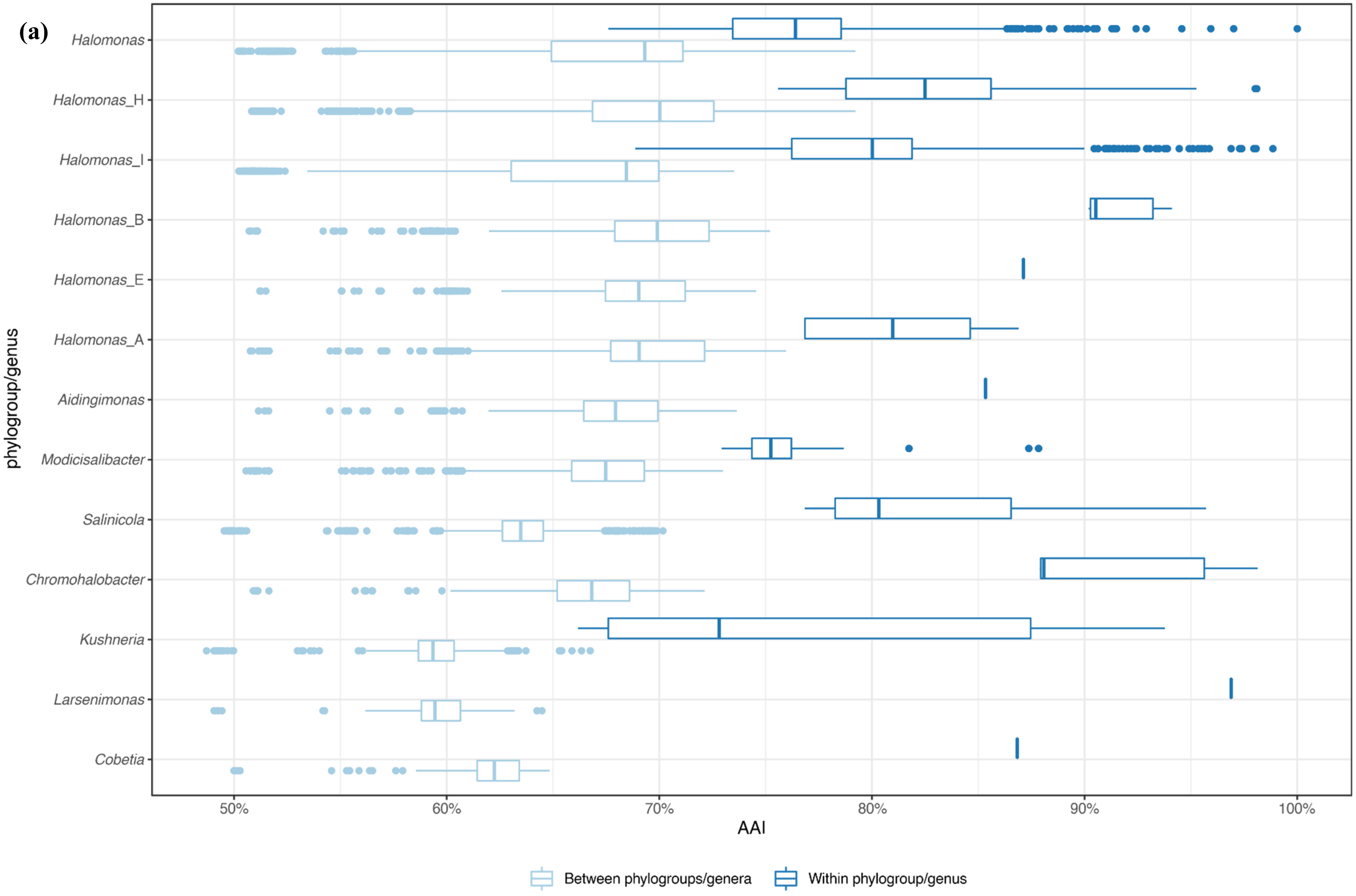

(a)
Halomonas
Halomonas_H
Halomonas_I
Halomonas_B
Halomonas_E
Halomonas_A
Aidingimonas
Modicisalibacter
Salinicola
Chromohalobacter
Kushneria
Larsenimonas
Cobetia
phylogroup/genus
50%
60%
70%
80%
90%
100%
AAI
Between phylogroups/genera
Within phylogroup/genus

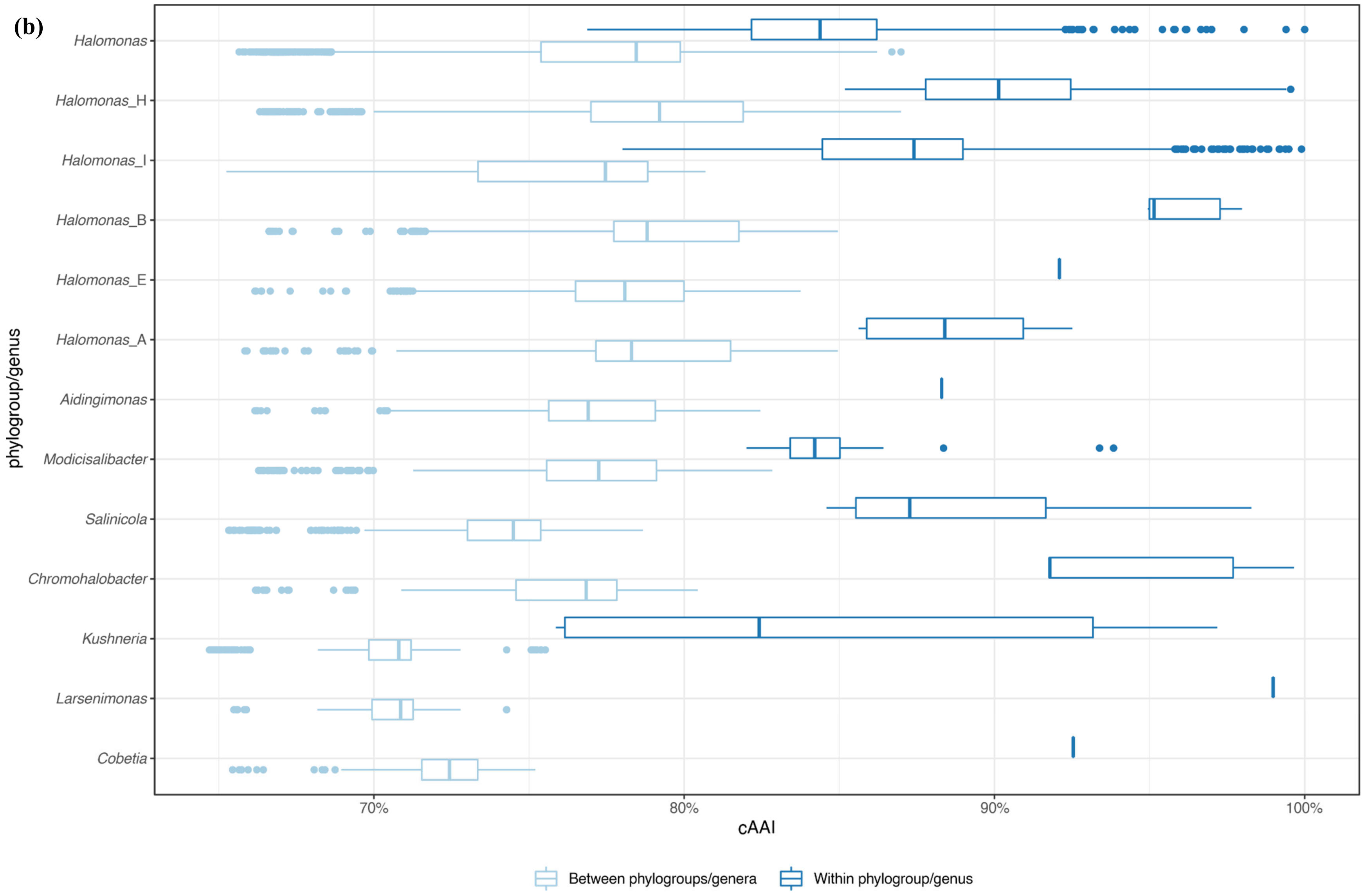

Supplementary Figure 2. Box plot of the pairwise intra-clade and inter-clade AAI (a) and cAAI (b) values for all the potential phylogroups/genera in the family *Halomonadaceae* according to *proposal II*.

(a)

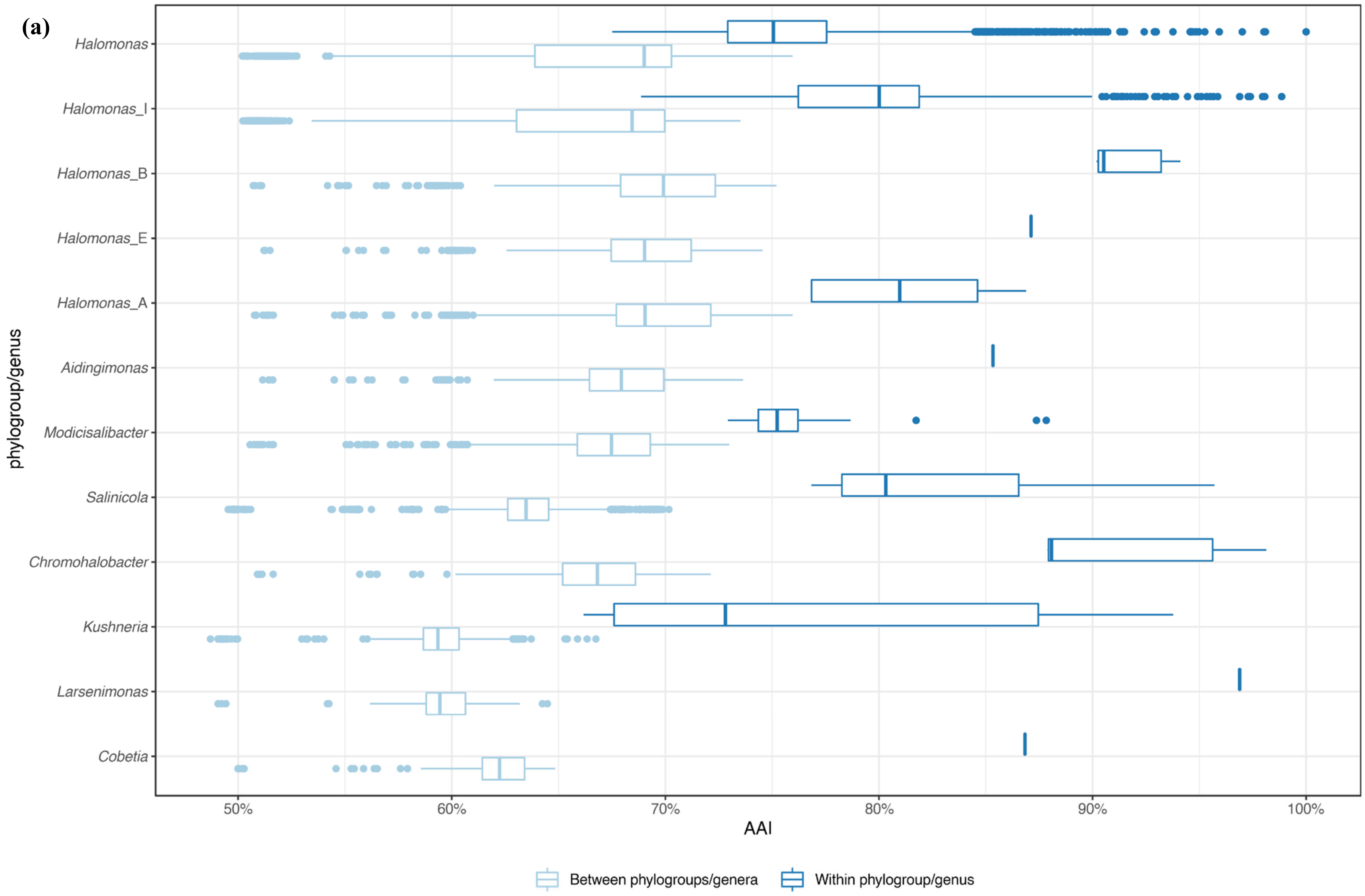

Halomonas
Halomonas_I
Halomonas_B
Halomonas_E
Halomonas_A
Aidingimonas
Modicisalibacter
Salinicola
Chromohalobacter
Kushneria
Larsenimonas
Cobetia
phylogroup/genus
50%
60%
70%
80%
90%
100%
AAI
Between phylogroups/genera
Within phylogroup/genus

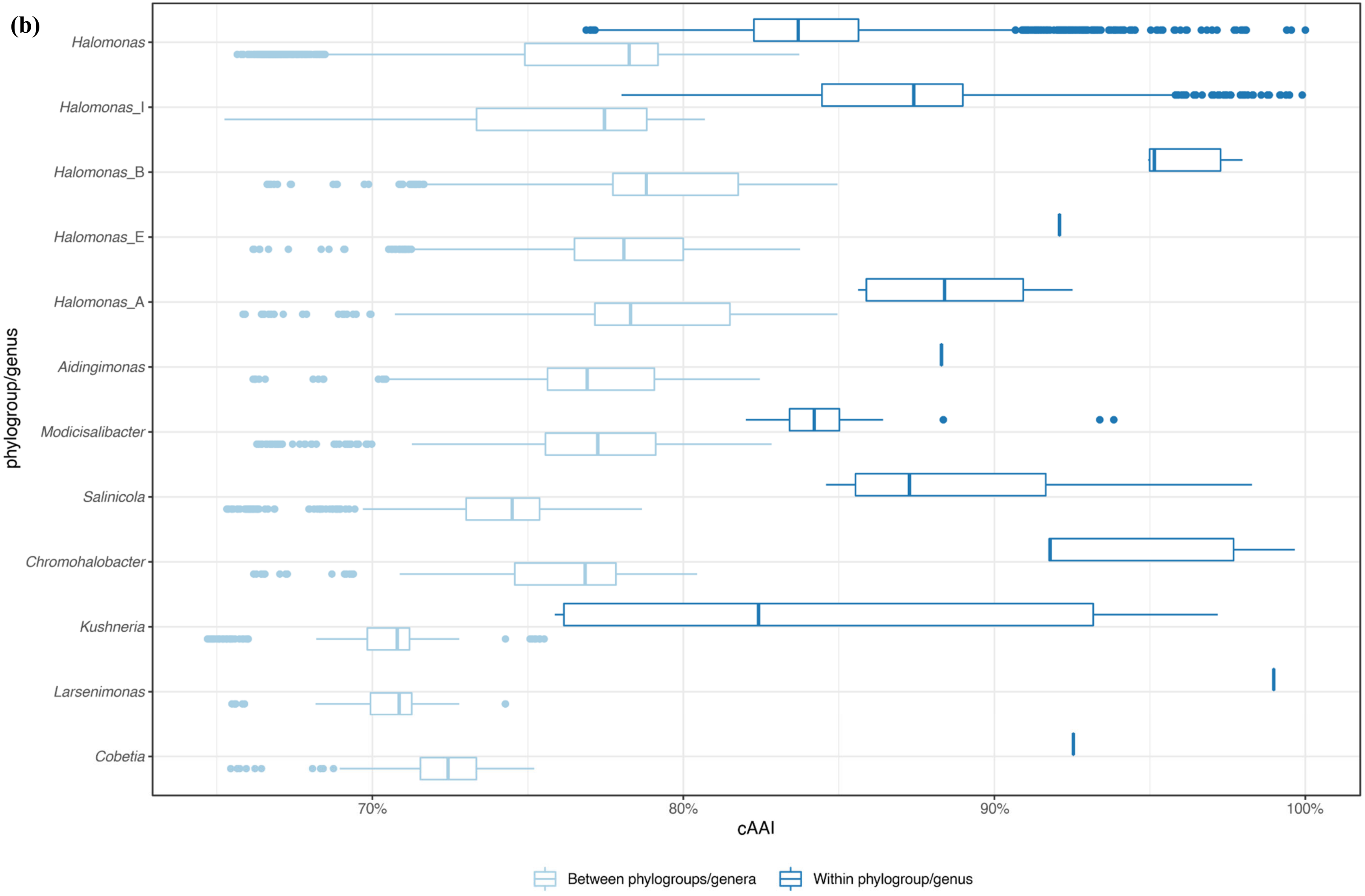

Supplementary Figure 3. Box plot of the pairwise intra-clade and inter-clade AAI (a) and cAAI (b) values for all the potential phylogroups/genera in the family *Halomonadaceae* according to *proposal III*.

(a)

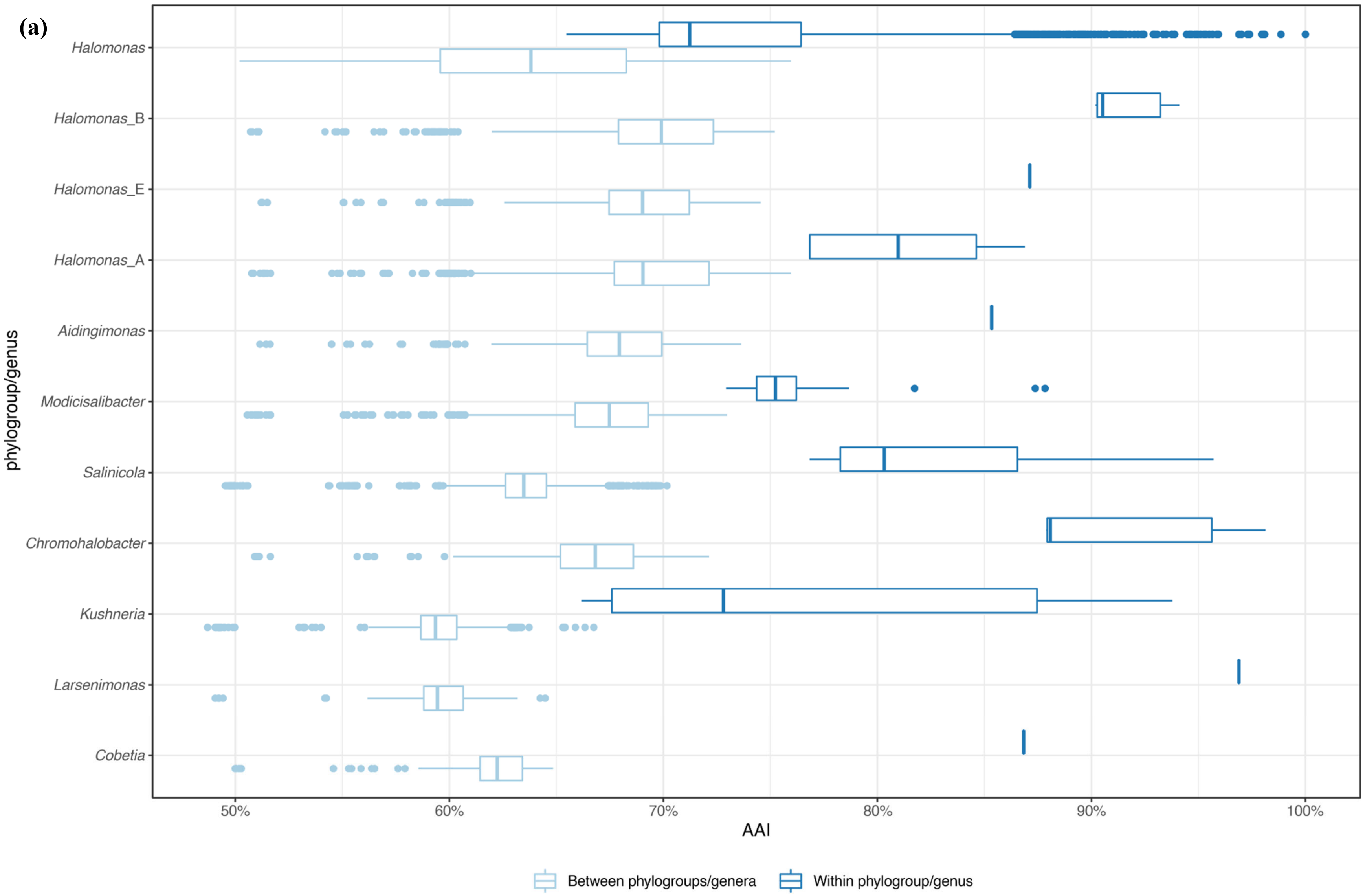

Halomonas
Halomonas_B
Halomonas_E
Halomonas_A
Aidingimonas
Modicisalibacter
Salinicola
Chromohalobacter
Kushneria
Larsenimonas
Cobetia
phylogroup/genus
50%
60%
70%
80%
90%
100%
AAI
Between phylogroups/genera
Within phylogroup/genus

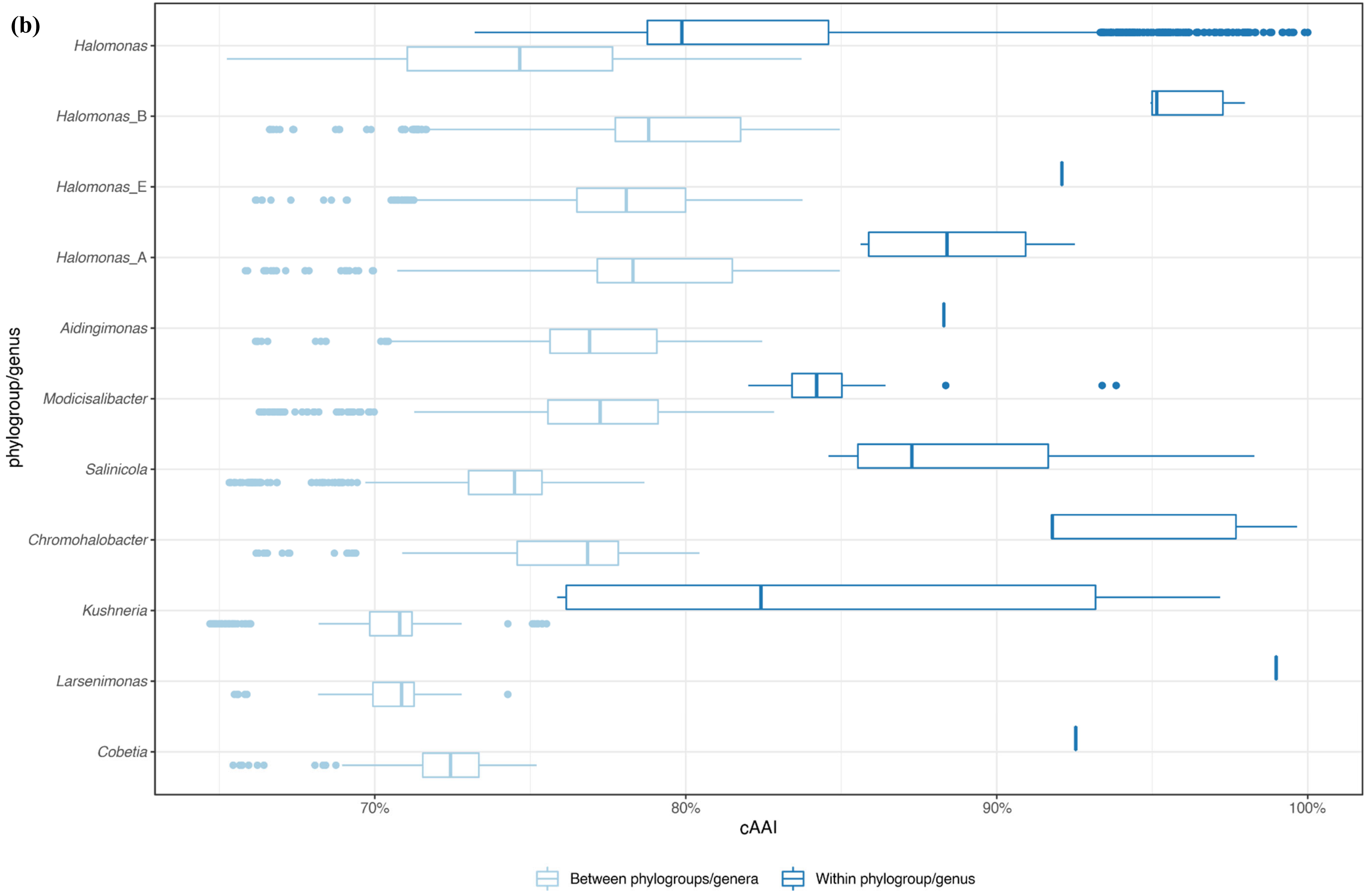

Supplementary Figure 4. Box plot of the pairwise intra-clade and inter-clade AAI (a) and cAAI (b) values for all the potential phylogroups/genera in the family *Halomonadaceae* according to *proposal IV*.

(a)

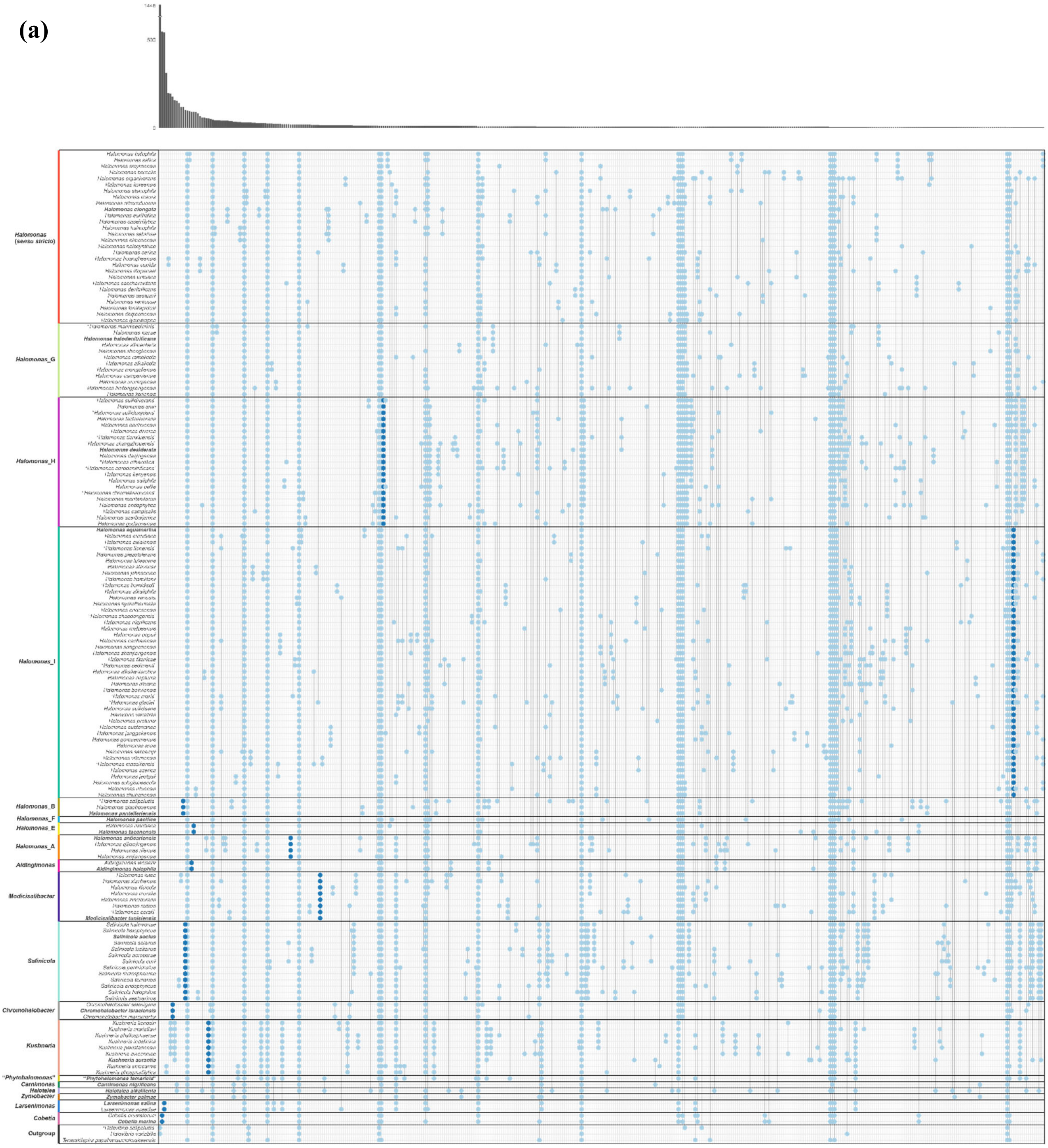
Halomonas (sensu stricto)
Halomonas_G
Halomonas_H
Halomonas_I
Halomonas_B
Halomonas_F
Halomonas_E
Halomonas_A
Aidingimonas
Modicisalibacter
Salinicola
Chromohalobacter
Kushneria
"Phytohalomonas"
Carnimonas
Halotalea
Zymobacter
Larsenimonas
Cobetia
Outgroup

(b)

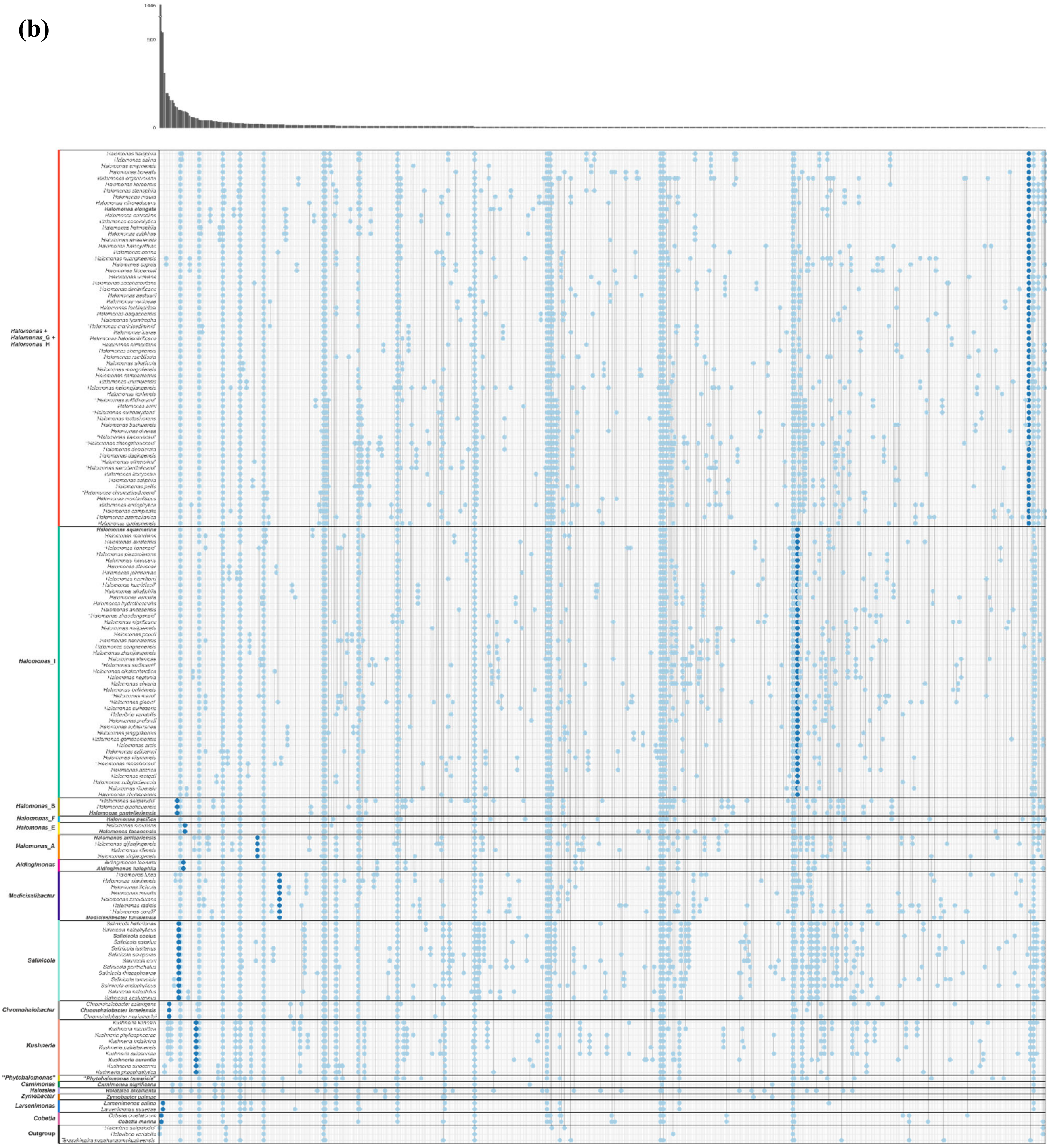
1446
500
0
Halomonas + Halomonas_G + Halomonas_H
Halomonas_I
Halomonas_B
Halomonas_F
Halomonas_E
Halomonas_A
Aidingimonas
Modicisalibacter
Salinicola
Chromohalobacter
Kushneria
"Phytohalomonas"
Carnimonas
Halotalea
Zymobacter
Larsenimonas
Cobetia
Outgroup

(c)

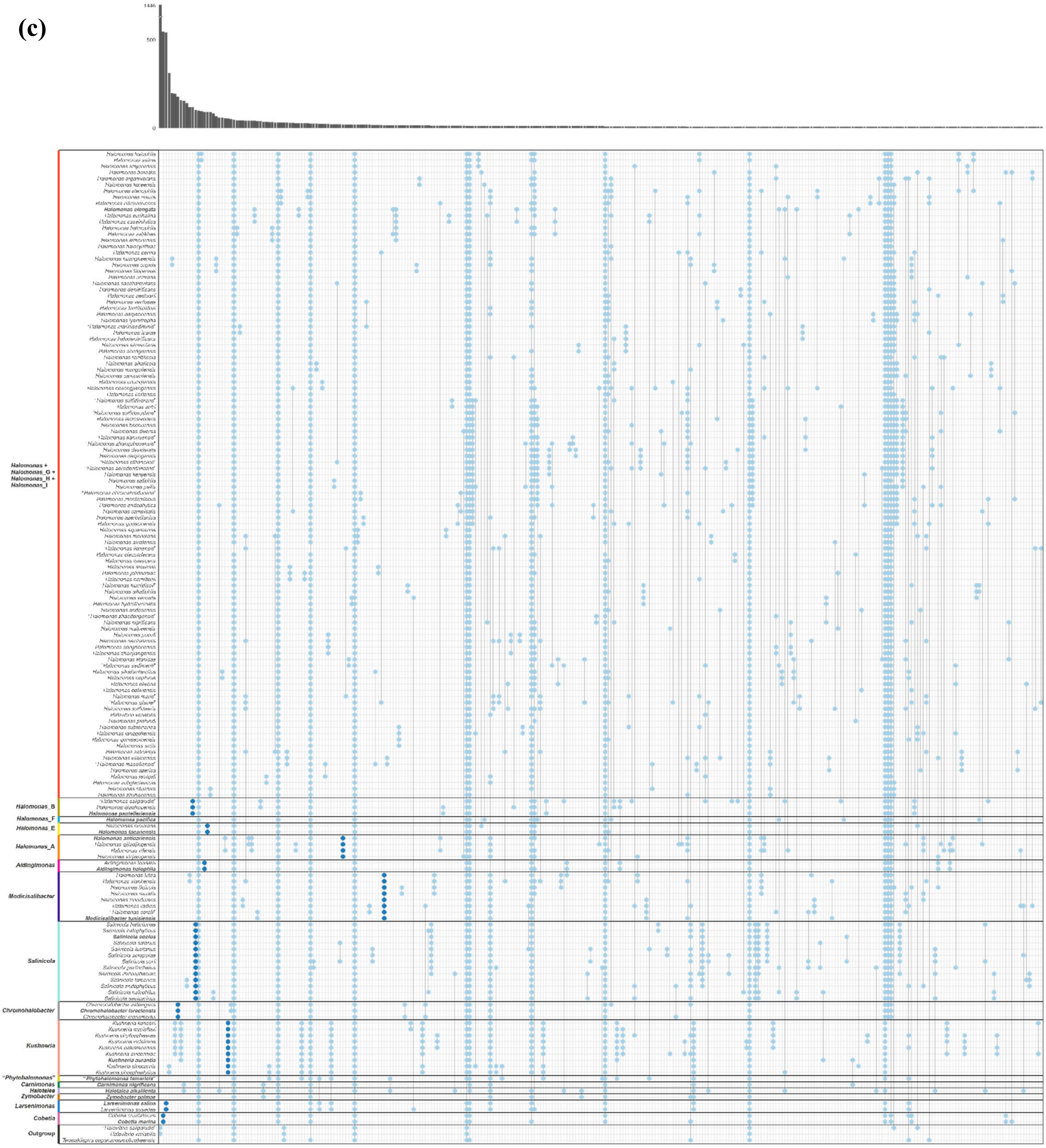

Supplementary Figure 5. Gene family presence/absence patterns inferred from the pangenome of the family *Halomonadaceae* arranged according to *proposal I* (a), *proposal III* (b), and *proposal IV* (c). Each column represents a gene family pattern, where presence is indicated with a dot in the corresponding species. The absolute number of gene families that conform to each pattern is visualized in the marginal bar plot at the top. Separations between phylogroups/genera are indicated with horizontal black lines and the representative species of each phylogroup/genus is highlighted in bold. Genes that were present in all genomes of a clade and in none of the genomes outside of that clade, denoted as "signature genes", are displayed in dark blue; other genes are shown in light blue. Patterns

of presence in a single species or all species are not shown. The species *Halovibrio salipaludis*, *Halovibrio variabilis*, and *Terasakiispira papahanaumokuakeensis* were not considered as members of the *Halomonadaceae* and were only used as an "outgroup".

2 Supplementary Tables

Supplementary Table 1. Type strains of species of the family *Halomonadaceae* and the genera *Halovibrio* and *Terasakiispira* whose genome sequences were available in public databases or obtained in this study*. The type species of each genus is highlighted in blue. NA, not available.

Species designation	Type strain	Accession No.	Additional NCBI Assembly accession. No.	Length (bp)	DNA G+C content (%)
***Aidingimonas halophila* Wang et al. 2009**	DSM 19219; KCTC 12885	GCA_900106955.1	GCA_014651655.1	3,886,483	58.18
***Aidingimonas lacisalsi* Guan et al. 2020**	XHU 5135	GCA_008079445.1	NA	3,953,408	58.07
***Carnimonas nigrificans* Garriga et al. 1998**	ATCC BAA-78	GCA_000526695.1	NA	2,726,123	56.20
***Chromohalobacter israelensis* (Huval et al. 1996) Arahal et al. 2001**	DSM 6768	GCA_000761475.1	NA	3,660,991	63.74
***Chromohalobacter marismortui* (ex Elazari-Volcani 1940) Ventosa et al. 1989**	DSM 6770	GCA_004364315.1	NA	3,553,220	61.71
***Chromohalobacter salexigens* Arahal et al. 2001**	DSM 3043	GCA_000055785.1	NA	3,696,649	63.91
***Cobetia crustatorum* Kim et al. 2010**	JO1	GCA_000591415.1	NA	4,049,952	57.52
***Cobetia marina* (Cobet et al. 1970) Arahal et al. 2002**	JCM 21022	GCA_001720485.1	NA	4,176,400	62.44
**"*Halomonas aerodenitrificans*" Wang and Shao 2021**	MCCC 1A11058	GCA_021404405.1	NA	5,082,661	64.03
***Halomonas aestuarii* Koh et al. 2017**	Hb3	GCA_001886615.1	NA	3,543,891	67.88
***Halomonas alimentaria* Yoon et al. 2002**	DSM 15356	GCA_009902005.1	NA	3,285,121	66.30
***Halomonas alkaliantarctica* Poli et al. 2011**	CRSS	GCA_004364445.1	NA	5,031,827	54.78
***Halomonas alkalicola* Tang et al. 2017**	CICC 11012s	SRX2700634	NA	3,639,102	67.57
***Halomonas alkaliphila* Romano et al. 2007**	DSM 16354	GCA_016107625.1	NA	4,095,328	52.58
***Halomonas almeriensis* Martínez-Checa et al. 2005**	CECT 7050	GCM10019084*	NA	2,815,138	63.54
***Halomonas andesensis* Guzmán et al. 2010**	DSM 19434	GCA_003989795.1	NA	3,907,727	52.06
***Halomonas anticariensis* Martínez-Cánovas et al. 2004**	FP35; DSM 16096	GCA_000409775.1	GCA_000428505.1	5,070,590	58.54
***Halomonas antri* So et al. 2022**	Y3S6	GCA_019430905.1	NA	4,389,574	64.25
***Halomonas aquamarina* (ZoBell and Upham 1944) Dobson and Franzmann 1996**	558	GCA_900110265.1	NA	3,497,205	56.73
***Halomonas arcis* Xu et al. 2007**	CGMCC 1.6494	GCA_900103865.1	NA	4,142,129	55.87
***Halomonas axialensis* Kaye et al. 2004**	Althf1	GCA_007163885.2	NA	3,619,799	56.78
***Halomonas azerbaijanica* Kazemi et al. 2021**	TBZ202	GCA_004551485.1	NA	4,576,545	67.52
***Halomonas azerica* Wenting et al. 2021**	TBZ9	GCA_013112225.1	NA	3,422,918	55.37
***Halomonas bachuensis* Xiao et al. 2021**	DX6	GCA_011742165.1	NA	4,701,666	63.63
***Halomonas boliviensis* Quillaguamán et al. 2004**	LC1	GCA_000236035.1	GCA_002265845.1	4,208,238	54.68
***Halomonas borealis* Diéguez et al. 2020**	ATF 5.2	GCA_004798905.1	NA	3,235,738	66.42
***Halomonas campaniensis* Romano et al. 2005**	5AG	GCA_014193375.1	NA	3,933,286	68.71
***Halomonas campisalis* Mormile et al. 2000**	A4; DSM 15413	GCA_031451595.1*	GCA_022341425.1	4,267,948	66.28
***Halomonas caseinilytica* Wu et al. 2008**	JCM 14802; CGMCC 1.6773	GCA_001662305.1	GCA_900109905.1	3,571,641	63.40

Halomonas cerina **González-Domenech et al. 2008**	CECT 7282	GCA_014192215.1	NA	4,168,933	65.72
**"*Halomonas chromatireducens*" Shapovalova et al. 2009**	AGD 8-3	GCA_001545155.1	NA	3,973,651	62.80
**"*Halomonas coralii*" Vidal et al. 2019**	362.1	GCA_004117855.1	NA	4,438,786	66.26
Halomonas cupida **(Baumann et al. 1972) Dobson and Franzmann 1996**	DSM 4740; NBRC 102219	GCA_900142755.1	GCA_007991155.1	4,957,973	59.64
Halomonas daqiaonensis **Qu et al. 2011**	CGMCC 1.9150	GCA_900109725.1	NA	3,716,433	64.01
Halomonas daqingensis **Wu et al. 2008**	CGMCC 1.6443	GCA_900108215.1	NA	4,737,211	64.87
Halomonas denitrificans **Kim et al. 2007**	DSM 18045	GCA_003056305.1	NA	3,758,327	68.74
Halomonas desiderata **Berendes et al. 1997**	FB2	GCA_011742915.1	NA	4,886,954	64.72
Halomonas diversa **Wang et al. 2021**	MCCC 1A13316	GCA_014931605.1	NA	4,490,931	62.90
Halomonas elongata **Vreeland et al. 1980**	DSM 2581	GCA_000196875.2	NA	4,061,825	63.61
Halomonas endophytica **Chen et al. 2018**	MC28	GCA_002879615.1	NA	4,980,457	62.14
**"*Halomonas ethanolica*" Wang and Shao 2021**	MCCC 1A11081	GCA_021404305.1	NA	4,573,332	64.47
Halomonas eurihalina **(Quesada et al. 1990) Mellado et al. 1995**	DSM 5720	GCA_031451635.1*	NA	3,842,566	63.02
Halomonas fontilapidosi **González-Domenech et al. 2009**	CECT 7341	GCA_014192285.1	NA	3,598,958	65.40
**"*Halomonas glaciei*" Reddy et al. 2003**	DD 39	GCA_013415125.1	NA	4,958,290	54.38
Halomonas gomseomensis **Kim et al. 2007**	DSM 18042	GCA_031451645.1*	NA	3,726,295	59.81
Halomonas gudaonensis **Wang et al. 2007**	CGMCC 1.6133	GCA_900100195.1	NA	4,166,643	64.94
Halomonas halmophila **(Elazari-Volcani 1940) Franzmann et al. 1989**	NBRC 15537	GCA_006540005.1	NA	3,253,825	63.70
Halomonas halocynthiae **Romanenko et al. 2002**	DSM 14573	GCA_000425725.1	NA	2,876,467	53.84
Halomonas halodenitrificans **(Robinson and Gibbons 1952) Dobson and Franzmann 1996**	DSM 735	GCA_000620045.1	NA	3,464,094	64.00
Halomonas halophila **(Quesada et al. 1984) Dobson and Franzmann 1996**	NBRC 102604	GCA_007989465.1	NA	3,511,261	68.13
Halomonas hamiltonii **Kim et al. 2010**	KCTC 22154	GCA_014651775.1	NA	3,932,903	60.11
Halomonas heilongjiangensis **Dou et al. 2015**	DSM 26881; 9-2	GCA_002879645.1	GCA_003202165.1	4,816,801	66.68
Halomonas huangheensis **Miao et al. 2014**	BJGMM-B45	GCA_001431725.1	GCA_000470745.1	4,757,486	58.53
**"*Halomonas humidisoli*" Liu et al. 2021**	WN018	GCA_002286955.1	NA	4,054,120	52.53
Halomonas hydrothermalis **Kaye et al. 2004**	Slthf2	GCA_011399095.1	NA	4,120,823	53.17
Halomonas icarae **Pandiyan et al. 2021**	D1-1; JCM 33602	GCA_009901955.1	GCA_031451795.1*	3,497,386	64.00
Halomonas ilicicola **Arenas et al. 2009**	DSM 19980	GCA_900128925.1	NA	3,960,292	63.16
Halomonas janggokensis **Kim et al. 2007**	DSM 18043	GCA_031451615.1*	NA	3,866,096	57.31
Halomonas jeotgali **Kim et al. 2011**	Hwa	GCA_000334215.1	NA	2,847,098	62.92
Halomonas johnsoniae **Kim et al. 2010**	KCTC 22157	GCA_014651795.1	NA	3,824,676	60.13
Halomonas kenyensis **Boltyanskaya et al. 2008**	DSM 17331	GCA_013697085.1	NA	4,420,996	63.76
Halomonas koreensis **Lim et al. 2004**	DSM 23530	GCA_031451675.1*	NA	3,817,634	69.71
Halomonas korlensis **Li et al. 2008**	CGMCC 1.6981	GCA_900116705.1	NA	4,038,005	62.51
Halomonas lactosivorans **Ming et al. 2020**	KCTC 52281	GCA_003254665.1	NA	4,360,527	66.65
**"*Halomonas lionensis*" Gaboyer et al. 2014**	RHS90	GCA_002087295.1	NA	3,646,653	55.92
Halomonas litopenaei **Xue et al. 2018**	SYSU ZJ2214	GCA_003045775.1	NA	4,815,616	64.13

***Halomonas lutea* Wang et al. 2008**	DSM 23508	GCA_000378505.1	NA	4,533,090	59.06
***Halomonas lutescens* Wang et al. 2016**	CGMCC 1.15122	GCA_014640815.1	NA	3,704,792	55.99
***Halomonas lysinitropha* Ramezani et al. 2020**	3(2)	GCA_902500215.1	NA	3,830,613	64.81
***Halomonas malpeensis* Kämpfer et al. 2018**	YU-PRIM-29	GCA_020622355.1	NA	3,607,821	63.76
**"*Halomonas marinisediminis*" Zhao et al. 2020**	204	GCA_004347715.1	NA	3,679,791	64.18
**"*Halomonas maris*" Qiu et al. 2021**	QX-1	GCA_013371085.1	NA	4,522,183	54.39
**"*Halomonas massiliensis*" Seck et al. 2016**	Marseille-P2426	GCA_900155385.1	NA	3,435,644	58.35
***Halomonas maura* Bouchotroch et al. 2001**	CECT 5298	GCM10019085*	NA	4,383,943	67.87
***Halomonas meridiana* James et al. 1990**	ACAM 246; NBRC 15608	GCA_900129255.1	GCA_006540125.1	3,864,078	56.96
***Halomonas mongoliensis* Boltyanskaya et al. 2008**	DSM 17332	GCA_031451685.1*	NA	3,549,079	67.71
***Halomonas montanilacus* Lu et al. 2020**	PYC7W	GCA_003336675.1	NA	4,785,753	62.87
***Halomonas muralis* Heyrman et al. 2002**	DSM 14789	GCA_900102945.1	NA	4,138,450	61.87
***Halomonas nanhaiensis* Long et al. 2013**	JCM 18142	GCA_003990185.1	NA	4,029,604	54.43
***Halomonas neptunia* Kaye et al. 2004**	CECT 5815; DSM 15720	GCA_030409295.1*	GCA_019903445.1	4,927,646	55.01
***Halomonas nigrificans* Oguntoyinbo et al. 2018**	MBT G8648	GCA_002374315.1	NA	4,929,385	52.80
***Halomonas niordiana* Diéguez et al. 2020**	ATF 5.4	GCA_004798965.1	NA	3,681,432	61.10
***Halomonas nitroreducens* González-Domenech et al. 2008**	11S	GCA_003966155.1	NA	4,352,895	67.54
***Halomonas olivaria* Amouric et al. 2014**	TYRC17	GCA_004295565.1	NA	4,998,083	55.34
***Halomonas organivorans* García et al. 2004**	CECT 5995	GCA_014192055.1	NA	4,801,488	66.19
***Halomonas pacifica* (Baumann et al. 1972) Dobson and Franzmann 1996**	NBRC 102220	GCA_007989625.1	NA	3,850,829	67.19
***Halomonas pantelleriensis* corrig. Romano et al. 1997**	AAP	GCA_900102875.1	NA	4,402,189	63.90
***Halomonas pellis* Li et al. 2020**	L5	GCA_008297955.1	NA	4,346,535	63.55
***Halomonas piezotolerans* Yan et al. 2020**	NBT06E8	GCA_012427705.1	GCA_009660035.1	3,945,801	57.93
***Halomonas populi* Xu et al. 2021**	MC	GCA_003989825.1	NA	3,803,855	54.98
***Halomonas profundi* Wang et al. 2022**	MT13	GCA_019504685.1	NA	3,601,857	54.03
***Halomonas qiaohouensis* Wang et al. 2015**	DSM 26770	GCA_031451695.1*	NA	4,652,051	64.54
***Halomonas qijiaojingensis* Chen et al. 2012**	KCTC 22228	GCA_014651875.1	NA	4,768,215	60.83
***Halomonas radicis* Navarro-Torre et al. 2020**	EAR18	GCA_900961225.1	NA	4,648,490	64.94
***Halomonas ramblicola* Luque et al. 2012**	CECT 7896	GCM10019087*	NA	4,123,506	67.87
***Halomonas rifensis* Amjres et al. 2011**	CECT 7698	GCM10020179*	NA	4,826,549	62.19
***Halomonas rituensis* Gao et al. 2020**	TQ8S	GCA_003336665.1	NA	4,474,525	57.24
***Halomonas sabkhae* Kharroub et al. 2008**	CECT 7246	GCM10019089*	NA	3,464,089	63.91
***Halomonas saccharevitans* Xu et al. 2007**	CGMCC 1.6493	GCA_900116405.1	NA	3,681,289	67.06
***Halomonas salicampi* Lee et al. 2015**	BH103	GCA_013415105.1	NA	3,864,100	56.19
***Halomonas salina* (Valderrama et al. 1991) Dobson and Franzmann 1996**	DSM 5928	GCA_031451735.1*	NA	3,529,351	68.10
**"*Halomonas salipaludis*" Xing et al. 2021**	WRN001	GCA_002286975.1	NA	5,475,884	63.83
***Halomonas saliphila* Gan et al. 2018**	LCB169	GCA_002930105.1	NA	4,344,030	64.08
**"*Halomonas sedimenti*" Qiu et al. 2021**	QX-2	GCA_013416325.1	NA	5,057,830	54.34
***Halomonas shengliensis* Wang et al. 2007**	CGMCC 1.6444	GCA_900104135.1	NA	3,530,709	68.62
***Halomonas smyrnensis* Poli et al. 2013**	AAD6	GCA_000265245.2	NA	3,561,919	67.86

***Halomonas songnenensis* Jiang et al. 2014**	CGMCC 1.12152	GCA_003002925.1	NA	3,686,518	59.14
***Halomonas stenophila* Llamas et al. 2011**	CECT 7744	GCA_014192275.1	NA	4,338,385	67.59
***Halomonas stevensii* Kim et al. 2010**	S18214	GCA_000275725.1	NA	3,693,745	60.25
***Halomonas subglaciescola* Franzmann et al. 1987**	ACAM 12	GCA_900142895.1	NA	3,110,197	60.77
***Halomonas subterranea* Xu et al. 2007**	CGMCC 1.6495	GCA_900111305.1	NA	3,734,199	57.97
***Halomonas sulfidaeris* Kaye et al. 2004**	ATCC BAA-803	GCA_007182875.1	NA	4,480,770	53.74
**"*Halomonas sulfidivorans*" Wang and Shao 2021**	MCCC 1A13718	GCA_017868935.1	NA	4,572,976	64.23
**"*Halomonas sulfidoxydans*" Wang and Shao 2021**	MCCC 1A11059	GCA_017868775.1	NA	4,491,948	65.99
***Halomonas taeanensis* Lee et al. 2005**	BH539	GCA_900100755.1	NA	3,756,722	62.31
**"*Halomonas tianxiuensis*" Wang and Shao 2021**	BC-M4-5	GCA_009834345.1	NA	5,015,118	63.93
***Halomonas titanicae* Mann et al. 2010**	BH1	GCA_000336575.1	NA	5,339,792	54.58
***Halomonas urmiana* Khan et al. 2020**	TBZ3	GCA_005780185.1	NA	4,028,094	66.89
***Halomonas urumqiensis* Zhang et al. 2016**	BZ-SZ-XJ27	GCA_003028575.1	GCA_002879635.1	3,978,292	62.62
***Halomonas utahensis* Sorokin and Tindall 2006**	NBRC 102410	GCA_007991175.1†	NA	3,729,154	55.81
***Halomonas ventosae* Martínez-Cánovas et al. 2004**	CECT 5797	GCA_004363555.1	NA	4,021,263	67.46
***Halomonas venusta* (Baumann et al. 1972) Dobson and Franzmann 1996**	NBRC 102221	GCA_007989605.1	NA	4,273,738	52.62
***Halomonas vilamensis* Menes et al. 2011**	DSM 21020	GCA_031451755.1*	NA	3,472,275	55.16
***Halomonas xianhensis* Zhao et al. 2012**	CGMCC 1.6848	GCA_900113605.1	NA	4,357,925	61.31
***Halomonas xinjiangensis* Guan et al. 2010**	TRM 0175	GCA_000759345.1	NA	3,786,651	60.69
**"*Halomonas zhangzhouensis*" Wang and Shao 2021**	MCCC 1A11036	GCA_021404465.1	NA	4,413,900	63.32
***Halomonas zhanjiangensis* Chen et al. 2009**	DSM 21076	GCA_000377665.1	NA	4,058,777	54.51
**"*Halomonas zhaodongensis*" Jiang et al. 2013**	NEAU-ST10-25	GCA_013415115.1	NA	3,724,647	53.03
***Halomonas zhuhanensis* Gao et al. 2020**	ZH2S	GCA_009793355.1	NA	3,248,142	57.14
***Halomonas zincidurans* Xu et al. 2013**	B6	GCA_000731955.1	NA	3,554,760	64.41
***Halotalea alkalilenta* Ntougias et al. 2007**	DSM 17697	GCA_000621205.1	NA	4,465,828	64.77
**"*Halovibrio salipaludis*" Tang et al. 2021**	YL5-2	GCA_002286965.1	NA	3,495,096	62.10
***Halovibrio variabilis* Fendrich 1989**	DSM 3050	GCA_019903425.1†	NA	3,525,189	61.82
***Kushneria aurantia* Sánchez-Porro et al. 2009**	DSM 21353	GCA_000382245.1	NA	3,750,691	62.76
***Kushneria avicenniae* (Soto-Ramírez et al. 2007) Sánchez-Porro et al. 2009**	DSM 23439	GCA_900112585.1	NA	3,605,329	60.32
***Kushneria indalinina* (Cabrera et al. 2007) Sánchez-Porro et al. 2009**	DSM 14324	GCA_003385845.1	NA	3,752,699	60.60
***Kushneria konosiri* Yun et al. 2017**	X49	GCA_002155145.1	NA	3,584,631	59.10
***Kushneria marisflavi* (Yoon et al. 2001) Sánchez-Porro et al. 2009**	SW32; DSM 15357	GCA_002157205.1	GCA_003610515.1	3,667,185	59.05
***Kushneria pakistanensis* Bangash et al. 2015**	KCTC 42082	GCA_014652795.1	NA	3,519,858	59.70
***Kushneria phosphatilytica* Du et al. 2021**	YCWA18	GCA_008247605.1	GCA_001854625.1	3,624,619	59.13
***Kushneria phyllosphaerae* Navarro-Torre et al. 2018**	EAod3	GCA_900312995.1	NA	3,764,595	59.37
***Kushneria sinocarnis* Zou and Wang 2010**	DSM 23229	GCA_003633775.1	NA	3,510,707	64.11
***Larsenimonas salina* León et al. 2015**	CCM 8464	GCA_023736155.1*	NA	3,206,856	59.15
***Larsenimonas suaedae* Xia et al. 2016**	DSM 22428	GCA_031451775.1*	NA	3,221,104	59.54

***Modicisalibacter tunisiensis* Ben Ali Gam et al. 2007**	LIT2	GCA_020148005.1	NA	3,550,803	67.37
**"*Phytohalomonas tamaricis*" Liu et al. 2020**	R4HLG17	GCA_003012345.1	NA	3,623,746	55.07
***Salinicola acroporae* Lepcha et al. 2015**	LMG 28587	GCA_003206615.1	NA	4,372,580	63.67
***Salinicola aestuarinus* Fidalgo et al. 2019**	CPA62	GCA_003206645.1	NA	3,869,882	63.57
***Salinicola corii* Li et al. 2021**	L3	GCA_008298015.1	NA	4,655,154	62.60
***Salinicola endophyticus* Fidalgo et al. 2019**	CPA92	GCA_003206575.1	NA	4,355,764	65.84
***Salinicola halimionae* Fidalgo et al. 2019**	CPA60	GCA_003206065.1	NA	3,991,304	60.62
***Salinicola halophilus* de la Haba et al. 2010**	CECT 5903	GCA_003206155.1	NA	3,615,108	64.87
***Salinicola halophyticus* Fidalgo et al. 2019**	CR45	GCA_003206695.1	NA	3,899,358	62.19
***Salinicola lusitanus* Fidalgo et al. 2019**	CR50	GCA_003206045.1	NA	4,415,417	64.32
***Salinicola peritrichatus* Huo et al. 2013**	JCM 18795	GCA_003206715.1	NA	4,623,416	62.45
***Salinicola rhizosphaerae* Raju et al. 2016**	KCTC 32998	GCA_014652715.1	NA	4,215,262	62.72
***Salinicola salarius* (Kim et al. 2007) de la Haba et al. 2010**	DSM 18044	GCA_003206135.1	NA	3,883,667	62.53
***Salinicola socius* Anan'ina et al. 2008**	SMB35; DSM 19940	GCA_001937195.1	GCA_003206115.1	4,174,124	62.23
***Salinicola tamaricis* Zhao et al. 2017**	F01	GCA_003006155.1	NA	4,276,240	65.68
***Terasakiispira papahanaumokuakeensis* Zepeda et al. 2015**	PH27A	GCA_001709345.1	NA	4,048,029	51.26
***Zymobacter palmae* Okamoto et al. 1995**	IAM 14233; DSM 10491	GCA_003610015.1	GCA_000620025.1	3,028,543	56.06

†GCA_007991175.1 and GCA_019903425.1 assembly accession numbers, originally deposited as *Halovibio variabilis* NBRC 102410^{T} and *Halomonas utahensis* DSM 3051^{T}, respectively, have been renamed as *Halomonas utahensis* NBRC 102410^{T} and *Halovibio variabilis* DSM 3050^{T} because NCBI records need to be updated after the Opinion 93 of the Judicial Commission of the International Committee on Systematics of Prokaryotes (*Int. J. Syst. Evol. Microbiol*. 2014, 64:3588-3589).

Supplementary Table 2. General time-reversible *Q_core90* matrix empirically estimated for the family *Halomonadaceae* derived from multiple sequence alignments of the 189 orthologous proteins shared at least by 90% of the analyzed genomes.

	Ala	Arg	Asn	Asp	Cys	Gln	Glu	Gly	His	Ile	Leu	Lys	Met	Phe	Pro	Ser	Thr	Trp	Tyr	Val
**Ala**	0.233786																			
**Arg**	0.259842	0.690994																		
**Asn**	0.708963	0.065072	6.890623																	
**Asp**	1.025844	2.232401	0.265576	0.042301																
**Cys**	0.550176	3.138676	1.955817	0.293166	0.220165															
**Gln**	1.343883	0.146402	0.407337	6.36527	0.004364	3.145345														
**Glu**	1.613074	0.501067	1.932514	1.783841	0.833768	0.296601	0.575594													
**Gly**	0.149005	2.947408	6.266875	0.779749	1.279801	5.624027	0.153801	0.268577												
**His**	0.05988	0.043664	0.218696	0.008303	0.576097	0.012291	0.010968	0.021852	0.049568											
**Ile**	0.141434	0.252429	0.017043	0.005916	0.679336	0.549808	0.012476	0.036339	0.225057	3.164449										
**Leu**	0.320369	5.319202	3.11949	0.162706	0.099021	4.393515	1.073436	0.33599	0.407835	0.070407	0.047613									
**Lys**	0.356173	0.188882	0.292563	0.079614	0.308322	0.51702	0.063655	0.077833	0.114591	3.157079	4.29731	0.678078								
**Met**	0.103828	0.018018	0.020118	0.001982	1.479431	0.016861	0.005095	0.047566	0.381928	0.684511	1.682207	0.018159	0.504315							
**Phe**	1.419597	0.367406	0.120114	0.293684	0.113362	0.692395	0.322815	0.185497	0.484897	0.031343	0.282278	0.346058	0.173739	0.046112						
**Pro**	3.409608	0.906518	8.121313	0.784504	4.215241	0.723126	0.406483	2.655032	0.70123	0.109481	0.132816	0.527053	0.384308	0.198941	2.153613					
**Ser**	3.27041	0.423512	3.108245	0.317537	0.809193	0.629534	0.51942	0.177485	0.395095	1.484312	0.136144	1.584788	1.879172	0.054191	0.901151	6.843888				
**Thr**	0.075519	0.383705	0.031116	0.0001	1.274898	0.069258	0.006964	0.216244	0.139951	0.049377	0.532894	0.033748	0.342986	0.988606	0.030369	0.091646	0.042597			
**Trp**	0.044318	0.1614	0.372295	0.060382	1.606011	0.082013	0.024318	0.023833	6.566942	0.128108	0.067056	0.01834	0.12285	9.582161	0.02799	0.168483	0.08354	1.272554		
**Tyr**	1.947656	0.072127	0.095089	0.039081	0.90006	0.170255	0.219413	0.113411	0.068332	12.980321	1.510402	0.084347	1.940423	0.410875	0.137593	0.082025	1.376786	0.090405	0.234116	
**Val**	0.116548	0.082587	0.020251	0.062108	0.007583	0.044609	0.074261	0.061063	0.027428	0.044069	0.11311	0.025164	0.026816	0.032345	0.039161	0.063416	0.05134	0.01346	0.021082	0.073597

Supplementary Table 3. General time-reversible *Q_bac120* matrix empirically estimated for the family *Halomonadaceae* derived from multiple sequence alignments of the 120 marker proteins used to infer bacterial GTDB taxonomy.

	**Ala**	**Arg**	**Asn**	**Asp**	**Cys**	**Gln**	**Glu**	**Gly**	**His**	**Ile**	**Leu**	**Lys**	**Met**	**Phe**	**Pro**	**Ser**	**Thr**	**Trp**	**Tyr**	**Val**
**Ala**	0.469651																			
**Arg**	0.67945	0.870468																		
**Asn**	1.274181	0.168195	9.581589																	
**Asp**	1.845749	3.408407	0.176289	0.213704																
**Cys**	1.16018	4.577314	2.579948	0.48785	0.212758															
**Gln**	2.423003	0.272859	0.652489	12.190291	0.033016	5.008664														
**Glu**	2.913208	0.759585	3.644871	2.707763	0.925611	0.546359	0.890396													
**Gly**	0.411215	5.294252	13.264288	0.934021	1.526934	9.693225	0.206319	0.426149												
**His**	0.123893	0.107995	0.280662	0.040984	0.627694	0.120445	0.0396	0.07437	0.168089											
**Ile**	0.274751	0.468693	0.136373	0.070269	0.89809	1.033729	0.073118	0.073803	0.627238	4.940492										
**Leu**	0.654295	9.996391	5.680308	0.119604	0.072178	7.191918	1.323213	0.416746	0.599709	0.204385	0.169326									
**Lys**	0.708469	0.36724	0.234132	0.05406	0.569872	1.363355	0.137748	0.105163	0.315746	7.47646	12.243278	0.832224								
**Met**	0.231991	0.142474	0.121492	0.085031	1.15735	0.123741	0.015675	0.034133	1.346374	0.726138	2.85213	0.040143	1.154914							
**Phe**	3.020874	0.486501	0.343601	0.570864	0.136493	0.956663	0.529381	0.359011	0.796275	0.098488	0.435276	0.403959	0.278917	0.081981						
**Pro**	8.06038	1.688877	13.878564	1.323791	6.381869	1.475571	0.632082	4.14645	1.412912	0.100292	0.180845	0.935392	0.224325	0.293142	3.121852					
**Ser**	5.529476	0.702728	3.961256	0.528004	1.447142	1.185029	0.862507	0.281894	0.658427	2.281396	0.268477	2.543016	3.394494	0.320433	0.973376	13.861991				
**Thr**	0.318437	0.879008	0.0001	0.114611	2.332242	0.367233	0.297463	0.840866	1.063438	0.245812	1.066386	0.283997	0.862668	1.986271	0.568372	0.288211	0.806238			
**Trp**	0.152274	0.48363	0.547806	0.109096	2.252423	0.173825	0.093265	0.027503	11.70761	0.363052	0.207393	0.031554	0.361481	22.7005	0.014542	0.238629	0.248601	3.566545		
**Tyr**	2.986564	0.130322	0.10507	0.102399	1.648999	0.235018	0.367145	0.101973	0.20862	22.338463	2.231487	0.165258	4.205804	0.600833	0.36769	0.162872	2.463922	0.161373	0.234116	
**Val**	0.112709	0.071204	0.022633	0.06528	0.007585	0.047697	0.080504	0.06531	0.023343	0.050761	0.117336	0.031575	0.018139	0.031202	0.034613	0.057444	0.051186	0.007048	0.021309	0.08312
